# Modeling Behavior by Coastal River Otter (*Lontra Canadensis*) in Response to Prey Availability in Prince William Sound, Alaska: A Spatially-Explicit Individual-Based Approach

**DOI:** 10.1371/journal.pone.0126208

**Published:** 2015-06-10

**Authors:** Shannon E. Albeke, Nathan P. Nibbelink, Merav Ben-David

**Affiliations:** 1 Wyoming Geographic Information Science Center, University of Wyoming, Laramie, Wyoming, United States of America; 2 Warnell School of Forestry and Natural Resources, University of Georgia, Athens, Georgia, United States of America; 3 Department of Zoology and Physiology, University of Wyoming, Laramie, Wyoming, United States of America; University of Girona, SPAIN

## Abstract

Effects of climate change on animal behavior and cascading ecosystem responses are rarely evaluated. In coastal Alaska, social river otters (*Lontra Canadensis*), largely males, cooperatively forage on schooling fish and use latrine sites to communicate group associations and dominance. Conversely, solitary otters, mainly females, feed on intertidal-demersal fish and display mutual avoidance via scent marking. This behavioral variability creates “hotspots” of nutrient deposition and affects plant productivity and diversity on the terrestrial landscape. Because the abundance of schooling pelagic fish is predicted to decline with climate change, we developed a spatially-explicit individual-based model (IBM) of otter behavior and tested six scenarios based on potential shifts to distribution patterns of schooling fish. Emergent patterns from the IBM closely mimicked observed otter behavior and landscape use in the absence of explicit rules of intraspecific attraction or repulsion. Model results were most sensitive to rules regarding spatial memory and activity state following an encounter with a fish school. With declining availability of schooling fish, the number of social groups and the time simulated otters spent in the company of conspecifics declined. Concurrently, model results suggested an elevation of defecation rate, a 25% increase in nitrogen transport to the terrestrial landscape, and significant changes to the spatial distribution of “hotspots” with declines in schooling fish availability. However, reductions in availability of schooling fish could lead to declines in otter density over time.

## Introduction

Forecasting changes in species distributions, migration patterns, population dynamics, and resiliency in response to predicted alteration of global climate has been in the forefront of ecological studies for the past few decades [1,2,3,4,5,6,7]. These investigations range from correlative empirical studies to complex modeling, as well as combinations of the two [8,9,10]. For example, using empirical data on abundance, survival and habitat selection of polar bears (*Ursus maritimus*) [11,12], in conjunction with stochastic population models parameterized with sea ice loss based on global circulation models, Hunter et al. [13] projected a 0.8–0.94 probability of extinction of the Beaufort Sea population by the year 2100.

Evaluation of the effects of climate change on animal behavior is mostly limited to migration and breeding phenologies [4,6,14,15,16]. A notable exception is the study of gray wolves (*Canis lupus*) on Isle Royal, Michigan, where a relation between snow accumulation and social grouping was established. Hunting by larger wolf packs facilitated an increase in kill rates of moose (*Alces alces*). As a result, the moose population declined facilitating an increase in the growth of balsam fir (*Abies balsamea*) [17]. The paucity of empirical and modeling studies on the potential effects of climate change on animal behavior and cascading ecosystem responses [17,18] is surprising. Because individuals are the building blocks of inherently complex ecological systems [19] and provide a natural scale at which to measure biotic and abiotic interactions [20], this seems an appropriate level for climate change investigations. Individuals are limited behaviorally and physiologically and their responses may be more predictable and easier to model than that of a population [21]. Additionally, individuals respond to internal and external environments by seeking to maximize ‘fitness’ through adaptive behavior, leading to the emergence of system level properties [19,21,22].

Individual-based simulation models (IBM), which treat individuals as unique and discrete entities, have been used since the 1970’s [22]. These discrete entities have several unique characteristics, such as age, that change during the cycle of the model [22]. IBMs have several advantages over analytical and stochastic system models, including variability among individuals, local interactions, complete life cycles [19,23], and responses to previous and current states (i.e. Markovian dependencies). Also, an emergent property of an IBM is the overall system stochasticity, precluding the need to combine the effects of multiple variance components associated with dynamic system models [24,25]. Further, spatially explicit IBMs integrate individual responses with landscape heterogeneity by specifying the explicit location of entities and their spatial relationship to other landscape features [26]. These advantages allow testing of theory under many different conditions, an attribute typically not available in natural systems [19]. This attribute is especially desirable given the myriad of potential climate change scenarios.

Similar to seabirds [27,28,29,30], piscivory by coastal river otters (*Lontra canadensis*) provides a pathway for nutrient transport between sea and land [31]. Marine-derived carbon (C), nitrogen (N), and phosphorus (P) transported by river otters to terrestrial latrine sites (specific locations along the shoreline) can be several orders of magnitude higher than other nutrient inputs in this system [32,33]. Uptake of marine-derived nutrients (MDN) associated with river otter activity increases photosynthetic capacity of the overstory layer of coastal conifer forests [34].

Coastal river otters exhibit atypical social behavior compared with other mammals [35,36]. In this system males occur in large groups (3–18 otters) that increase foraging efficiency on schooling pelagic fish within the nearshore environment [35,36,37,38]. Group size depends on the availability and spatial distribution of these pelagic fish [35]. In contrast, female otters and some males remain solitary year round, foraging primarily on intertidal-demersal fish. These individuals occasionally join a male group to opportunistically forage on pelagic fish, most likely because schooling pelagic fish have a higher energy density than the intertidal-demersal ones [35,39].

In coastal Alaska, social otters frequently use specific latrine sites as communication centers, advertising group association and dominance [37,40]. In contrast, nonsocial otters visit numerous latrine sites at low frequency, likely facilitating mutual avoidance [37]. These behavioral differences among individuals are determined by otter demography (abundance and sex ratio) and the distribution of pelagic fish in the nearshore environment. Extant spatial and temporal variation in the availability of pelagic fish leads to shifts in otter-mediated nutrient flux from sea to land, potentially making this system highly sensitive to future climate change.

The nearshore environment of coastal Alaska supports a diverse fish community composed of two distinct groups: resident intertidal-demersal species and migratory pelagic species. The intertidal species, primarily *Cottidae*, *Scorpaenidae*, *Hexagrammidae*, *Cancridae*, together with invertebrates such as mussels (*Mytilus trossulus*) and crabs (*Metacarcinus gracilis*, *M*. *magister* and others), are a ubiquitous, non-migratory prey [35,37,41,42]. In contrast, *Salmonidae*, *Ammodytidae*, *Clupeidae*, and *Gadidae* arrive in the nearshore environment to spawn [35,43]. These schooling pelagic fish species typically begin spawning in early May and return to the open ocean or expire (salmon) by November [42,44,45]. The responses of intertidal species to future climate change are unknown [46]. Nonetheless, decadal surveys conducted in the Gulf of Alaska, as well as species specific studies, demonstrate that schooling pelagic fish, who are cold water specialists, disappear from shallow coastal areas accessible to river otters with increasing sea-surface temperatures [47,48,49]. Thus, warming temperatures may result in decreases in availability of these fish, leading to shifts in otter sociality and nutrient transport to the terrestrial landscape.

Our goal was to investigate the effects of variation in the availability of schooling pelagic fish on nutrient transport by coastal river otters via a spatially explicit IBM. We tested six simulation scenarios based on potential shifts to spawning patterns of schooling fish in relation to warming sea-surface temperatures. We compared model results with empirical data we collected in this system in 2006 and 2007 as well as those reported in previous studies [35,36,37].

## Methods

### Study Area

**Fig 1:**
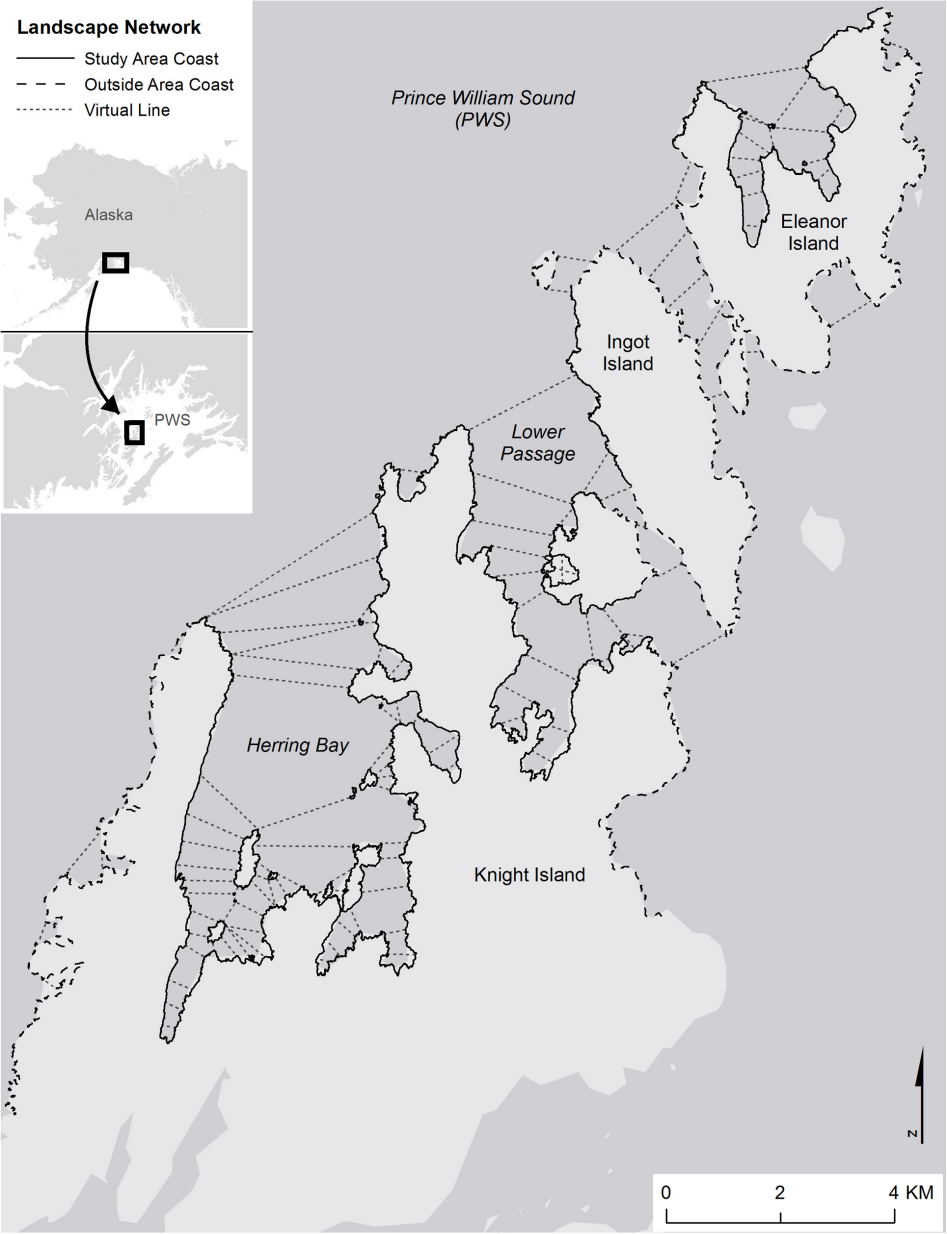
Map of the landscape network including the study area coastline, the additional outside area and the virtual lines connecting islands and bays. The study area coastline and the virtual lines are paths along which otters can move in the model. Outside area coast is not available to otters in the model.

The study area, of approximately 240 km^2^, encompassing 143 km of coastline, is located in the southwestern portion of Prince William Sound (PWS), Alaska and includes four islands: Knight Island (60.47 N, 147.75 W), Disk Island (60.49 N, 147.65 W), Ingot Island (60.53 N, 147.64 W) and Eleanor Island (60.55 N, 147.59 W; Fig 1). The region has a maritime climate with cool and wet summers followed by winters of deep snow accumulation [37]. The coastal landscape is typically snow-free from early May to early November. The structure of the coastline is primarily steep and rocky with some flat, low gradient beaches and numerous bays and inlets [50]. The coastal vegetation is predominantly old-growth forest of Sitka spruce (*Picea sitchensis*) and western hemlock (*Tsuga heterophylla*), with a well-developed under-story layer comprised of *Oplopanax horridus*, *Vaccinium spp*., *Menziesia ferruginea*, and *Rubus spp*. [31,34].

The bathymetric gradient of the nearshore environment is highly variable, ranging from near vertical to slopes of only a few degrees. The substrate is also variable with sizes ranging from large boulders to fine sediment [51]. Large tidal fluctuations in this habitat (annual maximum tide of 4.66 m and a minimum tide of -1.13 m [52]) greatly affect the vegetative community. Two kelp species (*Agarum cribrosum* and *Laminaria saccharina*) dominate within sheltered bays and less exposed coastline [41,53]. On exposed points, bull kelp, *Cereocystis luetkeana*, comprises the canopy and *Laminaria bongardiana* the understory [41,53]. Eelgrass, *Zostera marina*, grows on softer substrate usually found in inner bays [41,54,55,56]. The majority of the intertidal region is dominated by *Fucus gardneri*, interspersed with red and green algae [41,57,58].

### Landscape Network

As semi-aquatic mammals, coastal river otters rarely forage far from shore and even less commonly venture inland [51]. Therefore, their home range sizes are often calculated based on length of shorelines rather than area [51,59]. To model movements of river otters in our study we created a network of paths along the coast with corridors connecting individual islands or large bays on the same island [60]. This landscape network depicted the most probable paths used by otters (Fig 1).

To describe the terrestrial and marine habitats within the network, we obtained IKONOS 1m panchromatic stereo-pairs and 4m multispectral satellite imagery for the study area (GeoEye, Thornton, CO). Initially, using the Leica Photogrammetry Suite (LPS) within ERDAS IMAGINE (ERDAS, Inc., Norcross, GA), an existing Digital Orthro Quarter-Quad (DOQQ) aerial image was used as a reference for creating tie-points linking the two 1m panchromatic IKONOS stereo and multispectral images. From the georeferenced images, we derived three separate terrestrial datasets: 1) the coastline was digitized at a 1:1,500 view scale (245.3 km of shoreline), 2) supervised classification of five land cover classes (alder, conifer, muskeg, rock and water) was completed, and 3) a 10m digital elevation model (DEM) was derived [61]. Additionally, the marine portion of the landscape used by otters was created from bathymetric sounding points obtained through the National Geophysical Data Center [62]. We developed a 10m bathymetric model using the Inverse Distance-Weighting (IDW) algorithm.

We used the one marine and three terrestrial datasets to develop a suite of landscape variables describing the coastal and nearshore environment [63]. To the best of our ability, we chose metrics following Bowyer et al. [50,51] and Larsen [63]. Each metric was calculated for every 10m interval along the coastline (point-location). We employed Maximum Entropy [64,65] to estimate the probability (MEP) of each point-location used as an otter latrine [61]. During each simulation, MEP was used as a surrogate for otter habitat quality.

To complete the construction of the landscape network, we appended an additional 80.3 km of ‘virtual lines’ to the coastline network (Fig 1) to act as travel corridors between islands and across large bays [60]. The virtual lines were constructed through a multiple step process. First, we created Thiessen polygons using the 10m point-locations and converted into lines. For approximately every 2 km of coastline, the line connecting two islands or a bay was retained and the excess removed. The remaining virtual lines were slightly modified to create a straight line with only two vertices. Network Analyst tools within ArcGIS (ESRI, Redlands, CA) were used to identify network nodes and populate the adjacency table describing the connectivity of network edges. Portions of the landscape network were initially attributed as either within or outside the study area. The study area comprised 58% of the total available coastline within the landscape network.

### Simulations

We simulated six separate scenarios, based on abundance and spatial distribution of schooling pelagic fish, each replicated 100 times (Table 1). Each simulation was run using an hourly time step, beginning at 12:00am May 15 and running to 12:00am August 16 of the simulation year. Parameters included otter sex, activity-state (active or resting), number of hours in current activity-state, defecation-state (defecated or not), number of hours since defecating, satiation-state (fed on pelagic fish school or not), and spatial location (Table 2). Otter movements were simulated along the landscape network (Fig 1) which had three state variables assigned to each point-location (10m section of coastline): 1) the abiotic habitat quality (likelihood of being an otter latrine), 2) a radial-extent scaling factor (the ratio between the expected and actual network distances), and 3) potential pelagic fish spawning habitat (S1 Text).

**Table 1:** Model simulation of fish school scenarios (top) and sensitivity analysis (bottom) parameter values (mean [μ], variance [σ], and degree of adjustment in parentheses).

**Fish School Scenarios**		
**Scenario**	**Model Adjustment**	
School_100%	Baseline (from data collected in 1996–1999) and spawning habitat	
School_Random	Random placement with baseline availability	
School_75%	-25% school availability and spawning habitat	
School_50%	-50% school availability and spawning habitat	
School_25%	-75% school availability and spawning habitat	
School_None	No school availability and spawning habitat	
**Sensitivity Analysis**		
**Adjusted Parameter**	**Parameter Adjustment**	
	**Lower**	**Upper**
Movement Distance	μ = 847; σ = 1,558 (-10%)	μ = 1,035; σ = 1,904 (+10%)
Hrs Defecation	μ = 4.379; σ = 1.643 (-10%)	μ = 5.352; σ = 2.008 (+10%)
Hrs Active	μ = 1.418; σ = 1.379 (-10%)	μ = 1.734; σ = 1.685 (+10%)
Hrs Inactive	μ = 10.523; σ = 7.457 (-10%)	μ = 12.861; σ = 9.115 (+10%)
Scent Distance	-0.002 (-0.001)	-0.004 (+0.001)
Scent Decay Rate	0.001 (-0.099)	0.2 (+0.1)
Visual (m)	25 (-25m)	75 (+25m)
Memory (m)	500 (-500m)	1,500 (+500m)
Activity State (fish)	1.5 (-0.5)	2.5 (+0.5)
Defecation (fish)	1.5 (-0.5)	2.5 (+0.5)

Each simulation scenario was replicated 100 times.

**Table 2:** Parameters, associated values and statistical distributions (for random value selection) used for simulation initialization and model run-time for all scenarios, unless altered for the sensitivity analysis (Table 1).

Parameter	Value	Distribution	Description
*Model Initialization*			
Otter Density	55–78	Uniform	Minimum—Maximum 95% CI from density estimates [76]
Fish Schools	40–98	Uniform	Minimum—Maximum number of fish schools [37]
Gender Ratio	0.69	-	Percent of males in the population [76,77,78]
Female 50% Core Home Range	4 (2)	Normal	Mean (SD), in km, of female 50% Core Home Range [71]
Habitat Quality Threshold	0.464	-	Threshold probability value predicting otter latrine site [61]
Activity Threshold	0.122	Bernoulli	Probability of otter being in active-state (ratio of mean Active:Inactive hours; S1 Text, S1 Dataset)
Active Upper CI	1.88	Uniform	Upper 95% CI value of mean active-state hours (S1 Text, S1 Dataset)
Inactive Upper CI	15.32	Uniform	Upper 95% CI value of mean inactive-state hours (S1 Text, S1 Dataset)
*Model Run-time*			
Movement Distance	941 (1731)	Normal	Mean (SD), in meters, of 8 telemetered otters (S1 Text, S2 Dataset)
Hours Between Defecating	4.865 (1.825)	Normal	Mean (SD) hours between defecation events for captive otters in a 24hr period [79]
Hours in Active-state	1.433 (1.393)	Normal	Mean (SD) hours of continuous activity of 8 telemetered otters (S1 Text, S1 Dataset)
Hours in Inactive-state	11.692 (8.286)	Normal	Mean (SD) hours of continuous inactivity of 8 telemetered otters (S1 Text, S1 Dataset)
Scent Distance Decay Rate	-0.003	Bernoulli	Parameter estimate in negative exponential equation (S1 Text eq. 5) calculating probability of detecting scent-mark given a distance
Feces Desiccation Rate	0.1	Bernoulli	Parameter estimate in exponential equation (S1 Text eq. 5) calculating expected amount of desiccation (unitless value) given the age, in hours, of the fecal deposit
Visual Perception Distance	50	-	Assumed visual distance, in meters, at which the otter is acutely aware of the biotic and abiotic conditions of its surroundings
Memory Perception Distance	1000	-	Assumed memory distance, in meters, at which the otter can perfectly recall the best (MEP) available habitat
Active/Inactive—Satiation Scaler	2	Bernoulli	A scaling factor that increases (if Active) or decreases (if Inactive) the probability that an otter will switch activity-states if it has foraged on a school of fish (S1 Text eqs. 3 and 4)
Defecation—Satiation Scaler	2	Bernoulli	A scaling factor to increase the probability of a defecation event if the otter has foraged on a school of fish (S1 Text eq. 1).
Number of Fish Schools/day	-	-	Number of fish schools interpreted from Brown et al. [44] and Blundell et al. [35] (S1 Text eq. 7)

Specific descriptions and model processes can be found in S1 Text.

In all simulations, we constrained female movements by delineating a 50% core area along the landscape network (i.e. ‘edge’), with a point representing the center of their home range acting as an attractant. That is, once a female ventured beyond the edge, her next movement oriented toward the central location. Female core areas were exclusive because empirical studies have shown that females have low spatial overlap and distinct core areas of use [59]. Male movements along the network were unconstrained in that we allowed male movements to overlap female core areas, as well as areas occupied by other males [59].

**Fig 2:**
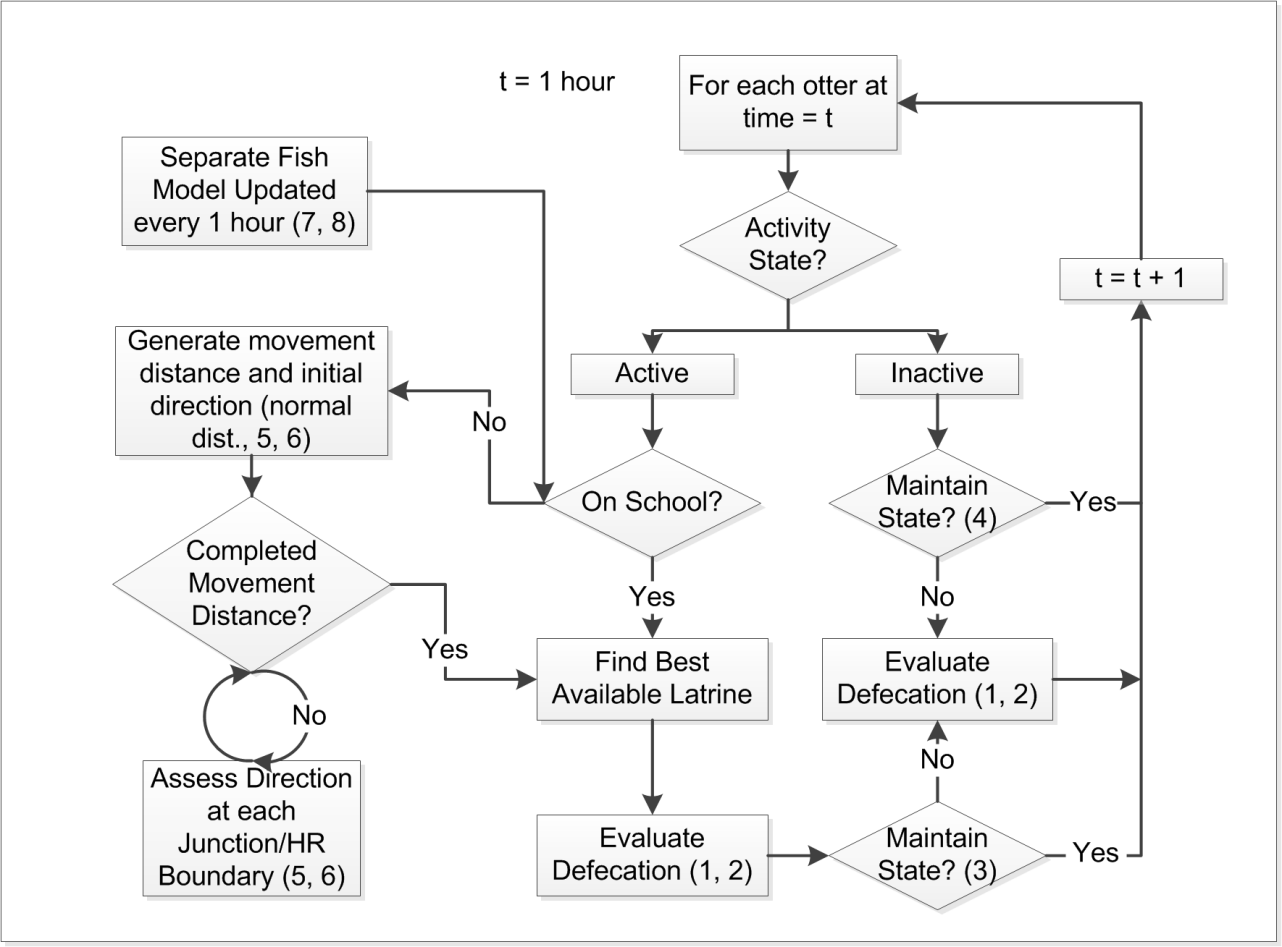
Model diagram describing the steps and decisions each simulated otter follows for each hour of the simulated period. Values within parentheses indicate model equations to determine individual otter choices (S1 Text).

The model simulates individual otter movement and behavior through foraging on both intertidal-demersal and schooling pelagic fish. Both male and female movements followed a Biased Correlated Random Walk (BCRW). Correlated random walks (CRWs) are those successive movements that have correlated directions [66]. In contrast, successive movements characterized by a consistent directional bias are termed BRCW [67,68,69,70]. In our case, the bias was composed of directional movements toward the central location of the female’s home range and the nearest latrine site within 1km of a successful encounter with a fish school for both sexes. Therefore, foraging behaviors were influenced by the presence of otter feces and/or fish schools. For example, an otter would continuously move along the landscape network within a search distance drawn from a distribution for each hour of activity, or until it reached within 50m of a fish school (Table 2). If an otter did not encounter a fish school in a given activity bout, it was assumed to have consumed intertidal-demersal fish. The model also accounts for post-absorbance resting and olfactory communication processes (Fig 2). At each time step, the activity-state of each otter was assessed with simulated behaviors occurring only when otters were active (Fig 2). The direction of movement along the network was determined by the detection of feces within 1km of the current location, simulating olfactory communication among group members (Table 2).

Each foraging event was followed by defecation behavior driven by the defecation-state, satiation-state and spatial location of the individual. For example, an otter had an increased probability of defecating at the nearest highest-quality latrine after a successful encounter with a fish school, simulating successful feeding, or after 8–9 hours have elapsed in which the otter is assumed to have fed on intertidal-demersal resources (Table 2). The maintenance of an individual current activity-state (active or inactive) was driven by the hours within the current activity-state and the satiation-state. For example, duration of resting at a static location (i.e., den) was a function of the time elapsed since entering the inactive-state and whether the otter successfully encountered a fish school in the preceding foraging bout (Fig 2; Table 2). For an exhaustive description of model process, following the protocol by Grimm et al. [23], see S1 Text.

Parameter mean values were held constant for the entire simulation given the conditions of the tested scenario, while for each individual at each time step the actual values were randomly drawn from the appropriate distribution (Tables 1 and 2; S1 Text). Response variables included defecation rate (proportion of hours each individual experienced a defecation event during the simulation), hours of activity (number of hours in the active-state per otter), fish school foraging success (number of occurrences in which an otter successfully located a fish school), social group interaction (percent time within a social group; group size), home range size (calculated as the length of shoreline), and coastline use (total number of fecal deposits per latrine site; S1 Text). We compared the results for these response variables for each schooling fish scenario (see below) to published empirical data [35,36,37,51,71,72] and those we collected in 2006 and 2007 in the same area.

### Schooling Fish Scenarios

To test the effects of potential climate change, we simulated six scenarios in which the availability and spatial distribution of schooling pelagic fish varied (Table 1). In the first scenario (Schools_100%), the hourly availability of fish schools to foraging otters was drawn from the maximal range of observed densities [35,44] based on the daily spawning patterns of these species as quantified in the late 1990s (S1 Text; eq. 7 and 8). The hourly landscape position of these schools was determined by point-locations characterized by spawning habitats with a depth ≤ 3m [73,74]. In the second scenario (Random_100%), availability was similar to the first scenario, but the spatial location was not limited by spawning habitat. In the remaining four scenarios, the availability of fish schools declined in 25% increments to 0% (Schools_75%, Schools_50%, Schools_25%, School_None) with locations constrained to spawning habitat. These scenarios were based on predictions that with increasing sea-surface temperatures, the abundance of spawning pelagic fish will decline in the nearshore environment [47,48,49].

### Model initialization

The density of otters within the study area was previously estimated to range between 0.28 to 0.8 otters/km of coastline (40–115 otters) [75]. Recent abundance estimates of resident otters suggested a range of 55–78 animals for the same area [76]. Thus, at the beginning of each simulation, the number of animals within the study area was randomly drawn from a uniform distribution bounded by these values. Estimates of otter demographic parameters within the study area were then extrapolated to the entire landscape network. For example, the total number of otters in the simulation was calculated by adding individuals to the resident population by multiplying the randomly drawn abundance by 0.585, which represents the fraction of coastline outside the study area.

Prior to simulation, each otter was assigned as male or female based on sex ratio of 69% male, 31% female, derived from previous studies [36,76,77,78]. The activity-state was assigned using a Bernoulli trial with ‘Active’ probability calculated as the ratio of average hours Active:Inactive (1.43:11.69; Table 2; S1 Dataset). The number of hours at the current activity-state was randomly assigned using a uniform distribution bounded by 0 and the upper 95% CI (1.88:15.32; Table 2; S1 Dataset) for each activity-state, rounded to the nearest integer. Ormseth and Ben-David [79] found that captive otters defecate, on average, once every 4.9 hours. Thus, the number of hours since last defecating was randomly drawn from a uniform distribution bounded by 0 and 5.

The final initialization step for each otter was its placement onto the landscape. This step was performed separately for the study area and out-of-area portions of the network. Point-locations were filtered to include only habitat values > 0.464 MEP, reflecting habitat characteristics of latrine sites favored by otters [61]. Male placement was unconstrained. Female home ranges were randomly drawn from a truncated normal distribution (value > 0) based on 50% core-area length of 4km (± 2SD) of coastline [71], with the central point-location adjusted using the radial extent scaling factor (S1 Text). Female 50% core areas on the landscape network did not overlap [71,80].

The timing and magnitude of spawning migration of pelagic fish to the nearshore environment varies annually by species [35,44,45,81,82]. Because of this variation, we did not differentiate between species of schooling fish. Using georeferenced, aerially identified fish school data provided in Ben-David et al. [37], the number of fish schools within 100m of the coastline, during a one-day period, were counted for years 1996–1999. The minimum (40) and maximum (98) number of schools were used to set the bounds for a uniform distribution depicting the maximum number of schools available during each simulation. The timing of fish school entry into the simulation is described by eq. 7 (S1 Text). The initial landscape position of fish schools differed for the six scenarios.

### Sensitivity Analysis

To estimate the relative importance of model parameters on simulated otter behavior, we conducted a sensitivity analysis using the School_100% scenario. In this analysis, we adjusted input values of the following parameters: foraging movement distance, visual detection distance, hours between defecations, hours actively foraging, hours inactive, scent detection distance, scent decay rate of feces, latrine location memory (attraction to the highest-quality latrine within 1km), change in activity state following fish school encounter, and change in defecation frequency following fish school encounter (Table 2; S1 Text). For all parameters for which we had empirical means and standard deviations (SD), the input values were adjusted by ± 10% by multiplying the mean by 1.1 and 0.9, respectively. To calculate the new SDs we multiplied the adjusted means by the coefficient of variation (CV). For those relationships in which we relied on expert knowledge (i.e., latrine location memory, change in activity state following fish school encounter, and change in defecation frequency following fish school encounter; Table 2) we adjusted the input variables by 50% to ensure a range of all possible conditions.

### Empirical data

This study was conducted in the Chugach National Forest and authorized under a Special Use Permit #GLA832 (expires on 12/31/2016). This project did not involve the use of vertebrate animals and did not require authorization by Institutional Animal Care and Use Committee. In 2006, while surveying the study area shoreline we identified 320 active river otter latrine sites. Of these sites, we selected 100 for monitoring fecal deposition using a stratified random sampling. We first stratified sites by activity level (> 100 scats—high use, < 100—low use) and then by location within the study area (Herring Bay, Lower Passage, and Northwest Bay of Eleanor Island). We re-visited these sites nine times between May and August and counted all new deposits at each sampling visit [34,76]. To ensure that we only counted fresh feces, we marked all present ones with crafting glitter (Glitterex Corp., Cranford, NJ) and counted only unmarked deposits at every subsequent visit. In 2007, we again counted fecal deposits on the same 100 latrine sites during five visits between May and August. Using the total fecal counts per site per visit in each year we calculated the mean fecal counts per day (mean feces/day).

### Data Analysis

The data from each replicated simulation scenario were compiled into a single MS SQL Server database. Data, including spatial location, were stored for each otter and fish school for each simulated hour. To account for variability, data were initially summarized for each replication and then for the entire simulation scenario. We compared results from the six fish-school scenarios by evaluating overlap of 95% CI for defecation rate, hours of activity, fish school foraging success, percent time within a social group, group size and 50% home-range size by sex. Home range size was estimated using Brownian Bridges [83]. We also compared values generated by these scenarios with published empirical data [35,36,37,51,71]. Similarly, we calculated mean and 95% CI for fecal counts per day for each scenario and compared those to counts we obtained in 2006 and 2007 in the same area.

To assess changes to patterns of nutrient deposition on the terrestrial landscape from fish school availability and distribution, we used Detrended Correspondence Analysis (DCA) [84,85]. In this analysis the mean number of feces deposited on each 50m of shoreline was calculated for every simulation by scenario. For each simulation (100 replicates), we repeated the DCA comparing the similarity in fecal deposition between scenarios. We calculated the mean and 95% CI and evaluated their overlap using values extracted from the first two axes of each DCA.

To assess the relative importance of the various model parameters, we created a tornado diagram using mean feces/day as the response variable. The diagram displays the range of mean response values and their associated 95% CI relative to the mean and 95% CI of the School_100% scenario. All statistical analyses were performed using Program R 2.15.1 [86].

## Results

Simulation scenarios were successfully completed for twenty-six separate parameterizations, each replicated 100 times. Each simulation replicate required approximately 15 hours to complete. The data from all simulation scenarios were captured within an SQL Server database composing a total of 2,695,027,086 records.

### Comparison to Observed

Model results from all scenarios closely matched observed patterns from empirical studies (Table 3). For example, otter abundance for the entire landscape was within the range of estimated values from non-invasive genetic sampling in 2006 (Table 3) [76]. Similarly, maximum observed group size ranged from 9–11 individuals [35]; our models yielded a range from 7–15 (Table 3). In addition, the percent of time social females spent in mixed-sex groups was 77.5% based on empirical data [35] and ranged from 82.1–83.0% in our simulations (Table 3). Finally, the percent of simulated feces containing pelagic fish for School_100% and Random_100% was equivalent to observed frequencies (Table 3) [37]. The only discrepancy occurred in 50% core home-range size for males where model results were 2 to 4 times higher than observed (Table 3) [71].

**Table 3:** Comparison of empirical data and simulated model results for each schooling fish scenario (Table 1).

Metric	Empirical	Random_100%	School_100%	School_75%	School_50%	School_25%	School_None
Otter Abundance	131 (95–189)	111 (109–113)	111 (109–113)	112 (110–114)	111 (109–113)	110 (108–112)	112 (110–114)
Maximum Group Size	9–11	7–14	8–14	8–15	8–14	7–14	7–13
Mean Min Group Size	1.9 (0.5)	2 (0)	2 (0)	2 (0)	2 (0)	2 (0)	2 (0)
Percent in Mixed Sex Group	77.5, 38	82.2 (0.4), 27.8 (0.3)	82.9 (0.4), 26.7 (0.3)	83.0 (0.3), 26.5 (0.3)	82.2 (0.4), 27.0 (0.3)	82.1 (0.4), 27.3 (0.3)	82.1 (0.3), 26.6 (0.3)
Percent of Feces with Pelagic Fish	39.9	40.0 (0.01)	35.9 (0.01)	29.3 (0.01)	22.7 (0.01)	12.5 (0.01)	0 (0)
50% Core Home Range	4 (2.6), 10 (2.6)	3.5 (0.5), 22.4 (1.2)	2.8 (0.5), 19.5 (1.6)	3.5 (0.6), 21.1 (1.6)	3.6 (0.5), 24.2 (2.1)	4.1 (0.5), 30.6 (2.4)	4.6 (0.5), 40.3 (3.4)

Values within parentheses indicate 95% confidence interval (n = 100 for each simulation scenario). Values for Percent in Mixed Sex Group and 50% Core Home Range are sex-specific with female values listed first, followed by males.

**Fig 3:**
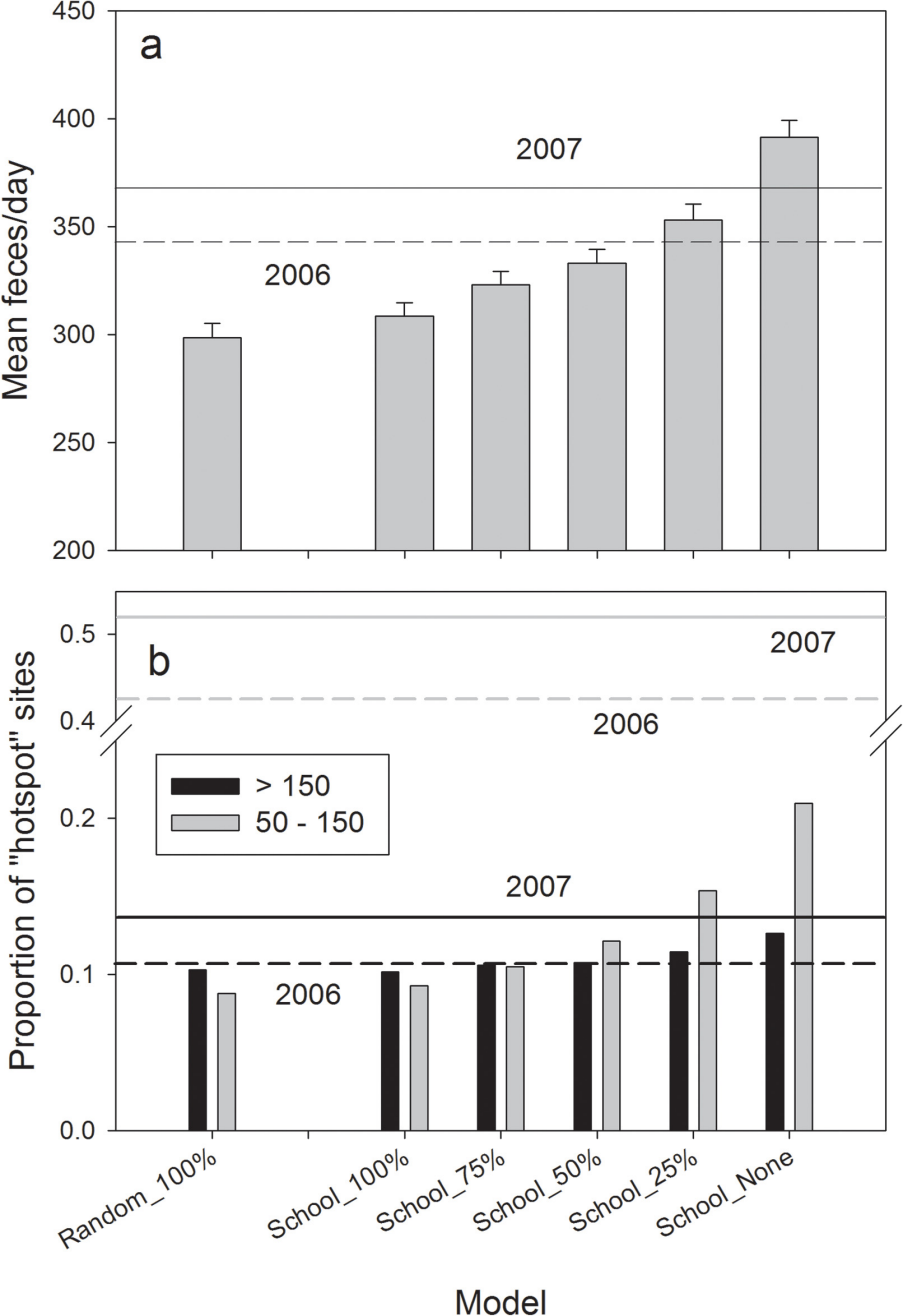
Observed and simulated fecal deposition rates and amounts for each schooling fish scenario; (a) mean feces per day with 95% confidence intervals, horizontal lines represent observed deposition rates; (b) proportion of summed 50m ‘windows’ (5 adjacent point-locations) along landscape network and observed proportion of latrine sites. (a) grey-dashed for 2006, grey-solid for 2007; (b) horizontal lines; dashed for 2006, solid for 2007. Grey shading represents locations having 50–150 feces, black shading for locations having more than 150 feces.

Mean feces/day, as estimated for all scenarios, ranged from 298.5 (±6.7 95% CI) to 391.5 (±7.7). This was within the range of observed rate of fecal deposition of 345.4 feces/day during 2006 and 365.6 feces/day in 2007 (Fig 3). While the proportion of “hotspot” deposition sites (> 150 feces) in the model was similar to that observed, the proportion of intermediate deposition sites (50–150 feces) was underrepresented in the model results (Fig 3).

### Model Sensitivity

**Fig 4:**
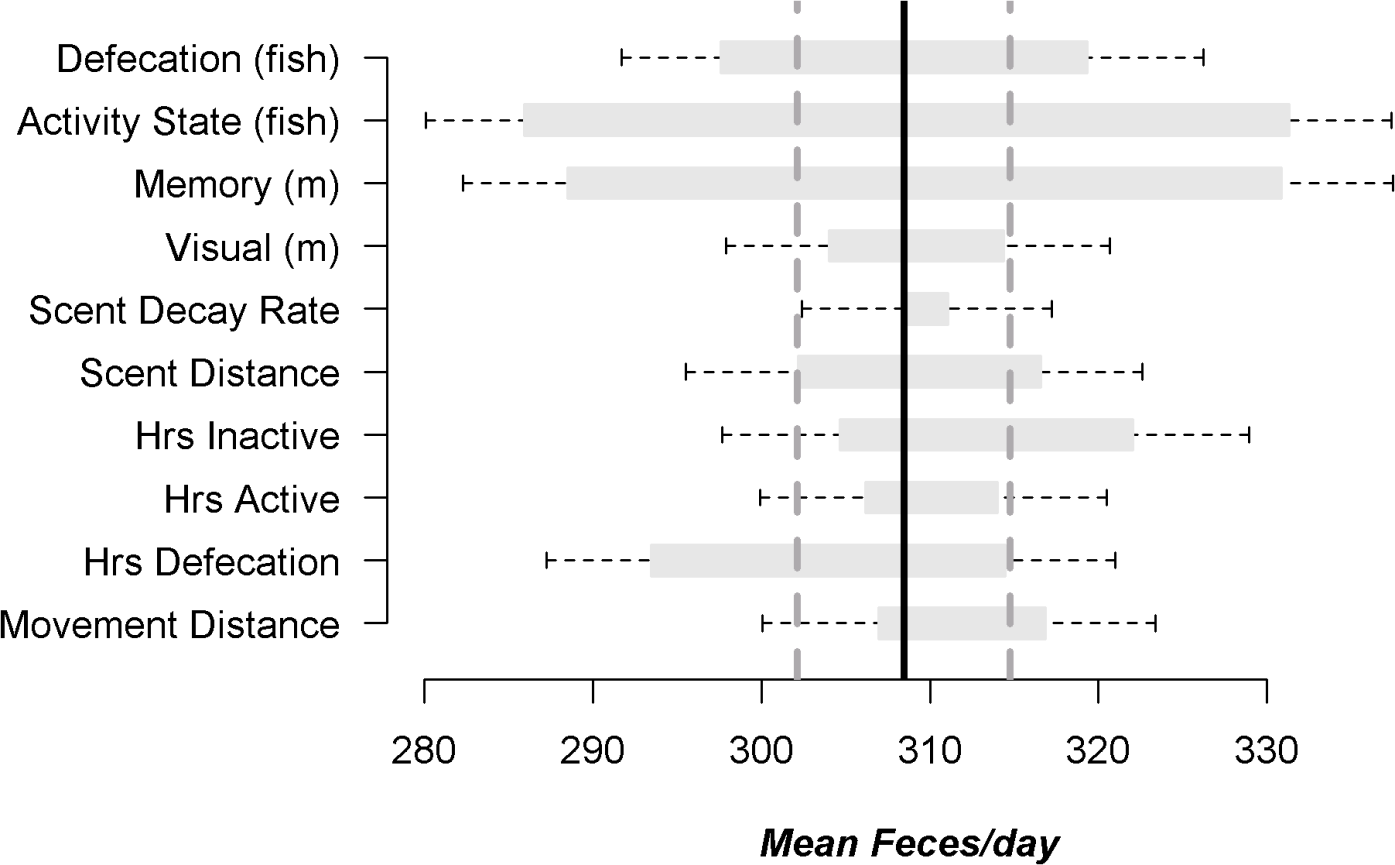
Diagram describing model sensitivity of mean feces/day, with 95% confidence intervals, as the response variable. The solid-black vertical line represents the mean (grey-dashed 95% confidence intervals) for the School_100% scenario (308 feces/day; baseline). For each adjusted parameter, the horizontal bar represents the mean values for two scenarios (lower and upper; see Tables 1 and 2). Each simulation scenario was replicated n = 100.

Mean feces/day was most sensitive to variation in activity-state following a fish school encounter and distance to nearest high-quality latrine (Activity State (fish) and Memory (m); Fig 4). The least influential parameter was Scent Decay Rate (Fig 4). Several parameters exhibited a skewed response to perturbation of the input values. Increasing the Hours Between Defecation events yielded a higher than expected reduction in the mean feces/day. Similarly, reducing the Hours Inactive disproportionately increased the mean feces/day (Fig 4).

### Schooling Fish Scenarios

**Fig 5:**
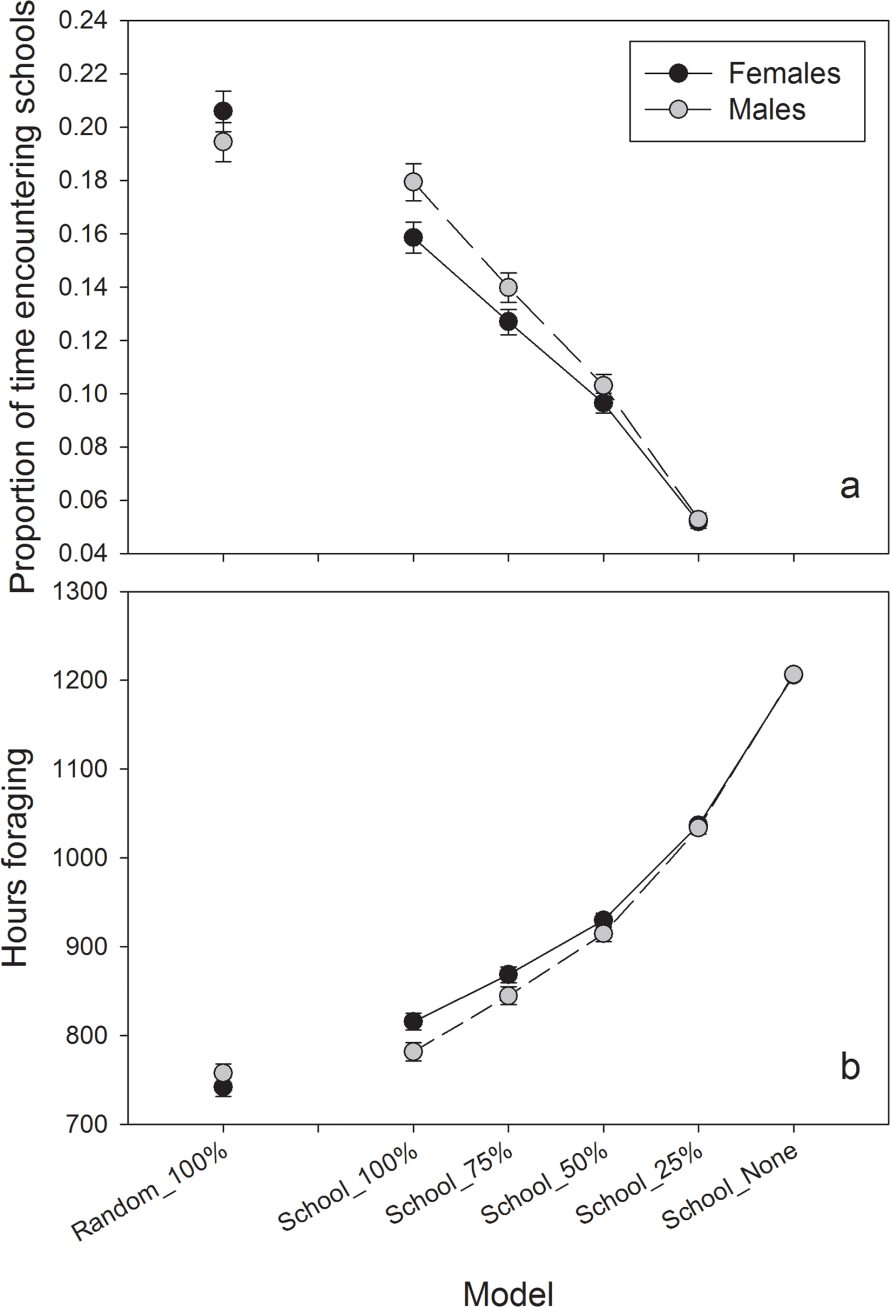
Simulated otter, by sex, forage success on schooling fish for each scenario; (a) proportion of total foraging effort, and (b) mean total hours spent foraging. Dark circles represent mean values for females and light circles for males, bars represent 95% confidence intervals (n = 100).

Changes in the availability and distribution of pelagic schooling fish resulted in significant changes to sex-specific behaviors of coastal river otters and associated fecal deposition rates. As expected, with a reduction in fish school availability, encounters with schooling fish by simulated otters drastically declined (Fig 5). Female encounters were significantly lower than males only for the School_100% and School_75% scenarios, suggesting that male otters assumed foraging strategies similar to females when fish schools became scarce (Fig 5). There was no sex-related difference in encounter rates when fish schools were randomly placed along the coastline. In this scenario, female encounter rates were significantly higher than when fish schools occurred in spawning habitat only (Fig 5). Concurrently, foraging time increased with decreasing fish school availability and followed similar trajectories for both sexes (Fig 5).

**Fig 6:**
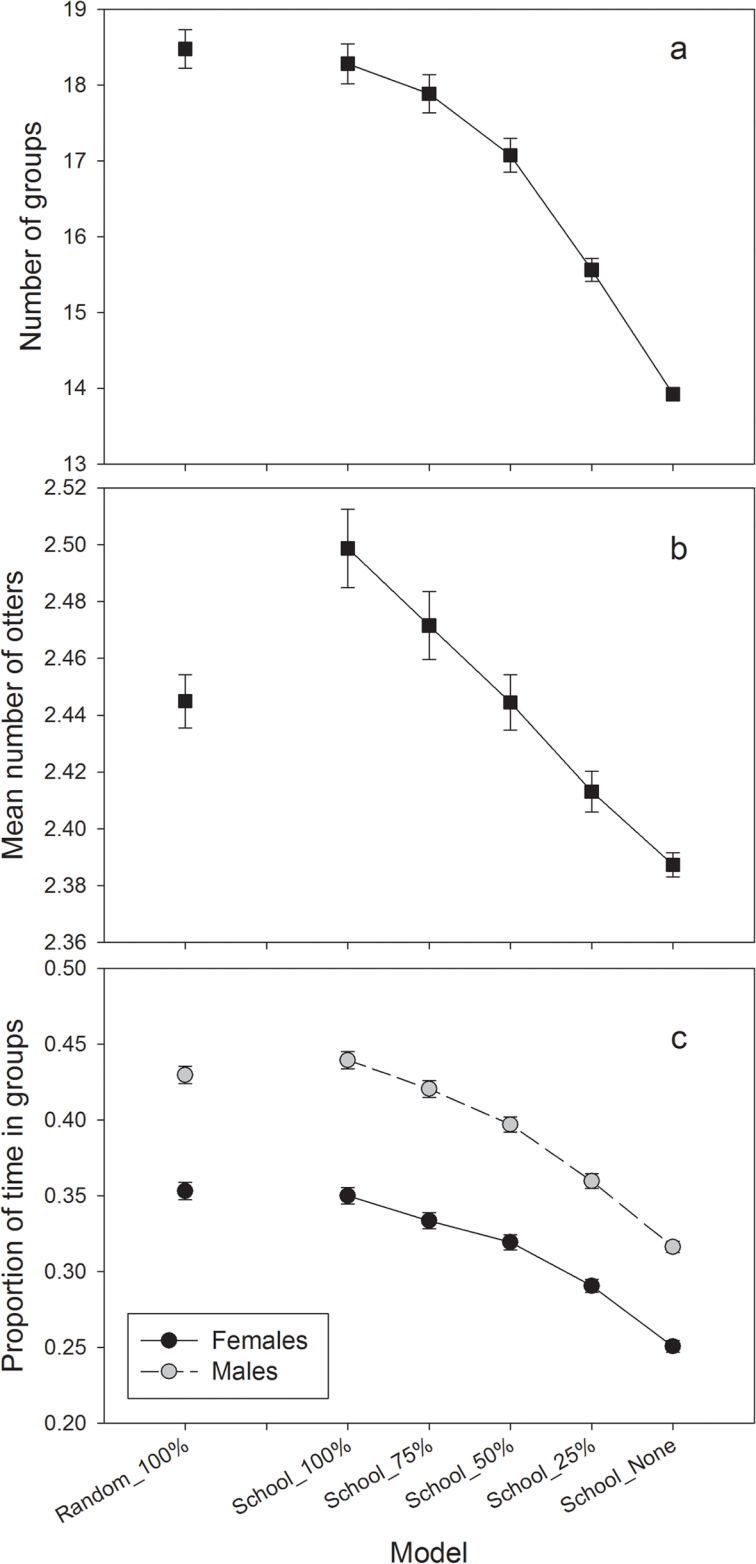
Simulated otter sociality for each scenario; (a) mean number of groups, (b) mean number of otters within a social group, and (c) mean proportion of time a female or male otter spent within a social group. Females represented by dark circle, male by light circle. Bars represent 95% confidence intervals (n = 100).

The mean number of otter-groups decreased as fish schools declined in availability, with the steepest rate of change occurring between School_50% and School_25% (Fig 6). Concurrently, the mean number of otters within social groups declined linearly (Fig 6). When fish schools were randomly placed along the coastline, the mean number of otters per group was significantly lower compared to School_100% and School_75% (Fig 6), although the difference amounted to only 2.4%. It is important to note that simulated otters had a tendency to aggregate into groups even without the presence of fish schools. The sexually dimorphic grouping behavior empirically observed in otters was replicated within the simulations, with males spending significantly more time in social groups than females for each scenario (Fig 6). As with group size and mean number of otters, time spent in groups declined with decreasing schooling fish availability, with no discernible difference between sexes.

**Fig 7:**
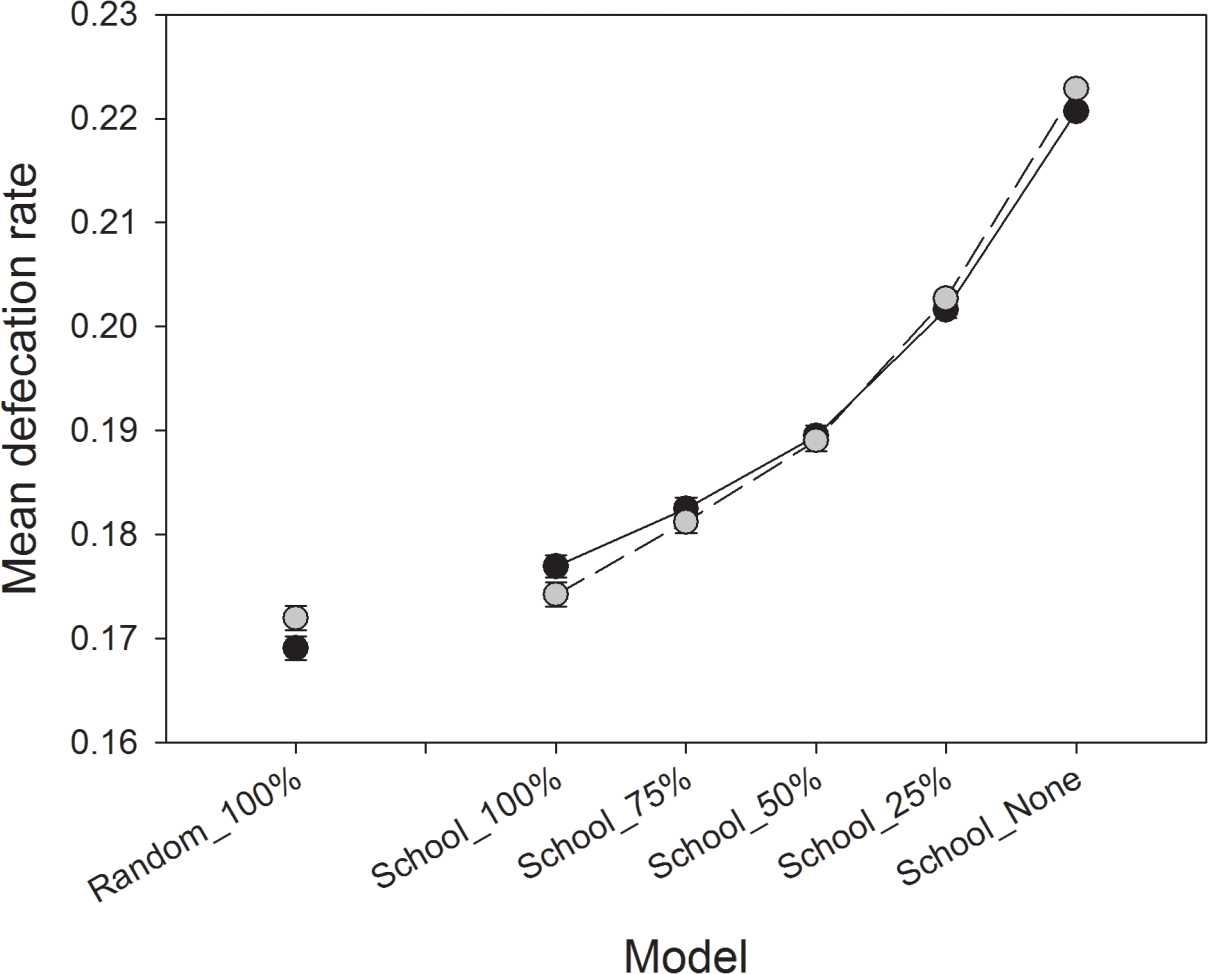
Mean defecation rate for simulated otters, by sex, for each scenario. Dark circles represent mean values for females and light circles for males, bars represent 95% confidence intervals (n = 100).

**Fig 8:**
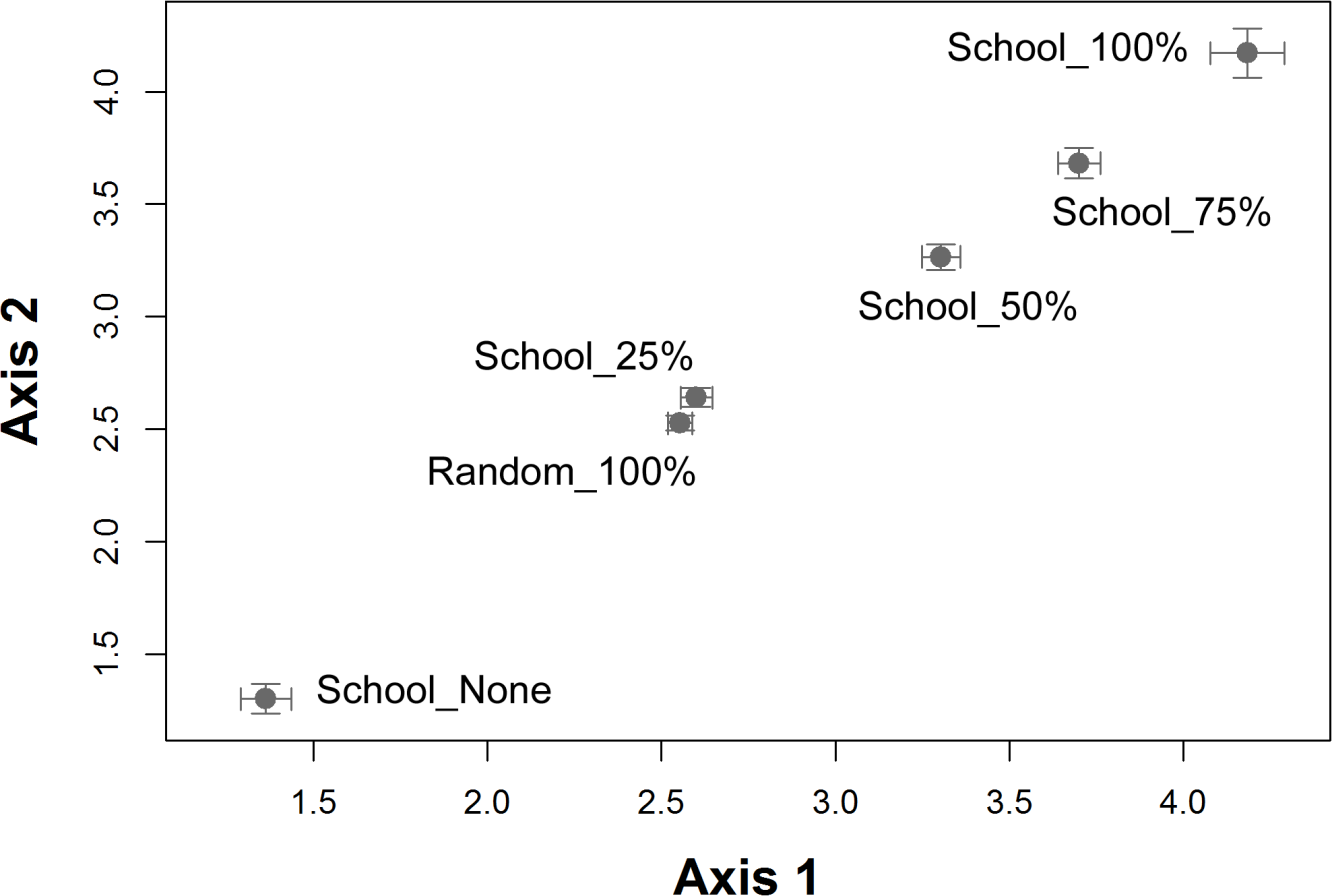
A scatterplot of the first two axes from a Detrended Correspondence Analysis of total fecal deposition along the simulated coastline for each fish school scenario. This plot helps to describe the spatial dissimilarity of fecal deposition. The bars represent 95% confidence intervals (n = 100 for each simulation scenario).

The mean defecation rate by otters significantly increased with decreasing availability of schooling pelagic fish, and was approximately 25% higher between School_None and School_100% (Fig 7). This translates into an increase of approximately 156 kg of MDN reaching terrestrial latrine sites annually (from estimated 580 kg School_100% to 736 kg School_None; assuming each deposit is equivalent to 5.15g of N as in Ben-David et al. [37]). In addition, the distribution of fecal deposition onto the landscape varied among scenarios, with an unexpected correspondence in the distribution of feces between Random_100% and School_25% (Fig 8). The proportion of “hotspot” sites with > 150 feces increased by 25% in response to declining availability of schooling fish (Fig 3). Concurrently, the proportion of sites with 50–150 feces increased by 125% although the distribution of “hotspots” on the landscape still deviated from observed patterns in both 2006 and 2007 (Fig 3).

## Discussion

The emergent patterns from the IBM we developed appeared to closely mimic observed otter behavior and landscape use, suggesting that the decision rules and model parameters we chose were a close approximation of conditions experienced by wild otters. For most response variables, except for male 50% core home-range and the proportion of “hotspot” sites, model estimates were within observed empirical ranges. The advantages of developing this IBM are clearly evident from the emergence of otter sociality without an explicit imposition within the model. However, these advantages may be overshadowed by model complexity and enormity of the resulting dataset. Having developed this model, we were able to forecast the potential effects of climate alteration via changes in schooling fish availability and demonstrated a significant change in the transport of MDN to the terrestrial environment in both quantity and distribution. Nonetheless, because our model was restricted to a single season, some of the emergent properties, such as the surge in fecal deposition rate, are likely a short-term response. In this model we did not account for a potential decline in demersal-intertidal fish given increased predation by otters in the absence of schooling pelagic fish; nor did we account for the likely decline in otter density over time. To fully understand the dynamics of this system under differing climate scenarios, this model will need to be extended to include multiple years and account for otter population vital rates.

The IBM approach is grounded in the belief that adaptive behaviors of individuals emerge as patterns at the system level [19]. To facilitate the expansion of IBMs in ecological theory, Uchmański and Grimm [87] proposed four criteria for a model to be considered an IBM: 1) the degree to which an individual’s life cycle is reflected; 2) the dynamics of individual resource use are explicitly represented; 3) real or integer numbers are used to represent population size; and 4) the extent to which variability between same age individuals is considered. We believe that our IBM meets these criteria: 1) although individual annual life cycle is not explicitly addressed, each individual’s daily activity is accounted for on an hourly basis. An individual uses stochastic rules, based on previous experience and states, to determine whether to forage or rest; 2) each individual may have the opportunity to locate and prey upon pelagic fish schools. Concurrently, pelagic fish schools are dynamically interacting with the physical environment instead of behaving in a static manner; 3) pelagic fish school densities are accounted for using real numbers and seasonal models. Otter density is estimated from a known distribution of the study area and; 4) each individual experiences both temporal and spatial variability throughout the modeling process. The variability may occur through spatial location (habitat quality), prey availability, and unique hourly experiences influencing decisions that affect current and future states. Thus, while we restricted this model to a single season and did not include otter vital rates in this simulation, it can be considered a special case of an IBM.

Our IBM produced separate social patterns for male and female otters in the absence of explicit rules of intraspecific attraction or repulsion. That is, foraging male otters were attracted to latrine sites containing fresh feces but not directly to each other, whereas females were attracted to the center of their core home ranges which did not overlap, indirectly creating repulsion. It appears that these simple rules are a self-organizing mechanism that generates sex-specific social groups in otters. Simulations of flocking and schooling behaviors in multiple species have been generated with relatively few rules of engagement, and groups were maintained without obvious fitness benefits [88,89,90]. Nonetheless, in such models the self-organizing rules include specific code for intraspecific synchronization and cohesion [88,89,90,91]. In contrast, in our modeled otter sociality and grouping behaviors emerged from foraging behavior loosely constrained by prey distribution on the landscape, suggesting that in some systems the formation of groups may be a by-product of resource distribution [92,93]. Nonetheless, while the number of simulated otter-groups and the time males and females spent in social groups declined with the reduction in schooling fish availability, sociality did not completely disappear with this resource. This agrees with observations of group formation in otters inhabiting freshwater systems. For example, Melquist and Hornocker [94] observed small groups of otters in Idaho and Serfass [95] recorded cooperative foraging in Canada. Thus it seems that in our model, as well as natural systems, other factors related to otter activity and landscape use foster aggregations.

To evaluate if other factors contribute to otter sociality, it will be imperative to assess whether simulated otters exhibited fidelity to certain groups. Although groups of wild river otters are formed at random with respect to kin [36,96], individuals exhibit high fidelity to their group [72]. Blundell et al. [72] observed that during the mating season (which ends prior to the initialization of our model in May) several adult males dispersed to adjacent areas, but returned to their home range and re-joined the group they previously left approximately a month later. Similarly, male otters that exhibited high levels of dyadic interactions while in captivity were found in close spatial proximity post-release [96]. Hansen et al. [96] hypothesized that familiarity was the process influencing group cohesion in wild otters and suggested that male otter pups may become familiar with neighboring male groups via olfaction when visiting latrine sites with their dames. If so, we would expect otters to produce individually distinct olfactory signals that are recognizable by others [97]. Rostain et al. [40] demonstrated that river otters are able to distinguish male and female feces as well as recognize the social status of animals relative to their own. These observations suggest that river otters excrete individualistic scent and are able to recognize the scent of others. Kean et al. [98] have shown that the feces of Eurasian otters (*Lutra lutra*) contain compounds unique to adults and juveniles as well as sex-specific ones. It is reasonable to assume that river otter feces contain similar compounds and that both species also have individually-unique scent.

Despite the high fidelity, river otter groups in the wild exhibit numerous fission-fusion events while foraging [99] and the structure of the social network varies through time [96]. In the future we will use results from our model to assess group fidelity, structure of the social network, and frequency of fission-fusion events of simulated otters and compare them to empirical data derived from radiotelemetry, non-invasive genetic analyses, and Encounternet proximity sampling [72,76,99]. Should we find that simulated otters show little group fidelity, we will develop an additional decision rule for the model creating attraction to familiar individuals based on individualistic scent. Such a rule may correct the discrepancy between observed and modeled male home range size.

Inherent to our modeling rules was the assumption that consumption of schooling pelagic fish confers fitness benefits, although no immediate advantage was observed among wild otters. Based on the observation that the number of offspring and relatives in the population did not differ between social and solitary animals, and otters adopting either strategy have similar size and condition, Blundell et al. [36] concluded that sociality did not produce fitness benefits. These authors hypothesized that the two social strategies (social and solitary), within the same population, persist because of large temporal fluctuations in the availability of schooling pelagic fish [36]. Testing of this hypothesis with empirical data will be impossible because the simultaneous collection of fish and otter data over an extended period of time will be impractical and inordinately expensive. Our results suggest that indeed when the availability of schooling pelagic fish declines, male otters forage and use latrines in a fashion resembling the behavior of females. Thus, once extended to include multiple years and account for otter population dynamics, our model could be used to test this and associated hypotheses.

Nonetheless, our model will require several adjustments before it can be extended. First, if simulated otters do exhibit high group fidelity, we will likely need to add a constraint on male movement to correct for the larger than observed male home ranges. Indeed, it is possible that our olfactory decision rule for male otters may have not been correctly formulated. The assumption that all feces are the same may need to be revisited because feces containing no pelagic fish may indicate resource depletion instead of resource availability [100]. Indeed, this may somewhat explain the drastic increases in male otter home-range sizes with decreasing availability of schooling pelagic fish.

Second, the model will require simplification because each single-season simulation took approximately 15 hours to complete. Our sensitivity analyses demonstrated that variation in Scent Decay Rate, Hours Active, and Visual (m) had relatively little influence on model outcomes, so these variables could potentially be made constant. It may also be possible to reduce the effect of the variable Memory (m), which forced the otters to travel to and potentially defecate at the nearest high-quality latrine within 1 km, rather than use any available latrine in their immediate vicinity. We created this decision rule because our field studies have shown that otters exhibit high fidelity to specific latrines, which are visited by multiple generations of otters [51]. Spatial memory has been documented from sharks to primates [101,102,103,104,105] and inclusion of such a parameter in models of animal movements has improved their accuracy [106,107,108]. From this perspective it is not surprising that this parameter had such strong influence on our results, although we may still capture the effects of memory by allowing otters to travel to the nearest latrine.

Indeed, this latrine selection rule probably caused the emergence of lower number of “hotspot” sites with 50–150 feces on the landscape as compared with our observed data. These emergent spatial properties of latrines were likely affected by the otter movement rule rather than the spatial placement of fish schools because the mean feces/day and the proportion of “hotspot” sites was similar between the Random_100% and the Schools_100% models. Only the actual location of used latrines was different between these two scenarios likely because foraging success increased when fish school locations were not restricted to specific spawning areas. Specifically, females experienced significantly higher success in locating fish schools than when these prey were patchily distributed. Because females were driven by different decision rules with regards to use of landscape, the distribution of latrines differed in this scenario.

Two processes may have influenced the lower fecal deposition rate we recorded in the Schools_100% and Schools_75% scenarios relative to values we observed in 2006 and 2007. First, we likely generated population estimates that were slightly biased low. We imposed a strong repulsion rule on females, basically that no two female core areas overlap. A relaxation of the rule may yield a slightly larger otter population. Also, we used the number of resident animals identified from non-invasive genetic sampling in 2006 within the study area [76] and then divided that value by proportion of modeled landscape that composed of the study area. The number of residents included adult animals only, whereas the wild population was augmented in July with pups emerging from natal dens [109]. Including recruitment in the model would have likely increased the overall population size and fecal deposition rate. In fact, the spatially-explicit simulated otter population could serve as an excellent source of information for designing fecal collection protocols that will yield unbiased estimates for the wild population. Because the location of each river otter during every hour of the simulation is known, and the location and timing of its fecal excretion is also recorded and saved, we could simulate any number of collection scenarios, and using mark-recapture modeling [110], test which protocol (in terms of number of collection days and number of latrines sampled) provides the least biased and most accurate abundance estimate.

Second, the higher than expected fecal deposition rate in 2006 and 2007 may have resulted from the fact that we simulated fish school availability with data collected in 1996–1999 [37,44], whereas we conducted the fecal deposition study nearly a decade later. Our observed fecal deposition rate suggests that in 2006 otters may have encountered a higher frequency of pelagic fish schools than in 2007. The mean sea-surface temperature in Prince William Sound was significantly different in June 2006 than in June 2007 (http://www.ndbc.noaa.gov/), likely increasing the availability of schooling fish for otter consumption during 2006 and decreasing their fecal deposition rate. Following this rational it is reasonable to assume that schooling fish abundance in our study area was higher in the 1990s than in the 2000s. A recent stock assessment for Pacific herring illustrated poor recovery of this fishery in coastal Alaska with oceanographic factors as one of three main contributors [111]. Thus, it is possible that schooling fish availability was lower than modeled when we collected the empirical data. This hypothesis can be tested by conducting dietary analysis on the feces we collected to generate the abundance estimates in 2006 and calculating the percent containing pelagic fish.

With a decline in schooling pelagic fish, otters may exhibit a two-pronged response. They will likely switch to more heavily prey on intertidal-demersal fish and decline in abundance. In our model we did not account for the density and distribution of intertidal-demersal fish explicitly, and assumed that benthic resources are uniformly distributed. Instead, the marine abiotic conditions that usually affect benthic fish in this system were included as variables within the model predicting latrine quality (MEP) [61]. Dean et al. [41] found differences in the distribution and abundance of benthic fish given characteristics of the marine environment. It may be an enlightening exercise to develop a model using only terrestrial variables for the otter latrine selection model and couple it with an intertidal-demersal fish habitat model. Such a model will allow us to assess the impact of varying benthic fish availability on otter behavior.

Our single-season model simulating the potential effects of climate change on nutrient transports from sea to land via the abundance of schooling pelagic fish and otter behavior resulted in several unexpected patterns. Foremost was the observation of elevated defecation rate and 25% increase in nitrogen transport to the terrestrial landscape. This occurred due to an increase in duration of the Active-State which reflected search of alternative prey and increased likelihood of visiting a latrine. This is clearly demonstrated within the sensitivity analysis in which Hours Inactive and Hours Between Defecation parameters had significant effects on mean feces/day. Also unexpected was the lack of decline in number of “hotspot” sites. While male group membership declined and their activity increased, their behavior in terms of attraction to latrines did not. A concurrent increase in nutrient deposition and the number of “hotspot” sites would have had significant implications to the vegetation at river otter latrines. Roe et al. [34] documented increased productivity of conifers growing at river otter latrines compared with those growing on non-latrine sites. Concurrently, enhanced river otter activity caused a reduction in shrub biomass and allocation of excess nitrogen to storage in shoots [34]. The shading from densely foliaged conifers also resulted in declines in understory plant diversity on latrines [112]. Thus enhanced latrine visitation and increased nitrogen fertilization could influence the terrestrial landscape in this system. Nonetheless, in response to reduced availability of schooling fish, male otters are likely to change their behavior. Reduction in schooling fish availability will likely result in increased predation on intertidal-demersal fish which occur at lower biomass [41] and have lower energy density than schooling pelagic fish [39]. Reduced resource availability will likely lead to declines in otter density over time. Indeed, otter density is substantially lower in ecosystems where food availability is diminished [113,114]. Thus, changes to terrestrial vegetation will be short lived. In future models we will assess the effects of climate change on nutrient transports in this system, by extending this model to include multiple years and account for otter population dynamics.

## Supporting Information

S1 Dataset Tab-delimited text file containing summarized observations of individual otter behavior over a 24-hour period. Column descriptions are as follows: IDNum (radio collar ID); MinOfStartTime (date/time describing the beginning time of the behavior); MaxOfStartTime (date/time describing the ending time of the behavior); DateGroup (integer identifying the unique set of observations for the individual otter); ActivityGroup (text identifying whether the otter was ‘Active’ or ‘Inactive’); SumHr (float summarizing the total number of hours in the current state for the individual otter); TotTime (float the total number of hours of observation).
(TXT)

S2 Dataset Tab-delimited text file containing summarized observations of individual otter movement rates in meters per hour. Column descriptions are as follows: Freq_StopID (text ID describing the individual collar ID and the observation ID in a ‘from-to’ format); Activities (text describing the specific otter behaviors at the ‘from-to’ locations. The codes: ‘AL’ = ActiveLand, ‘AW’ = ActiveWater, ‘AS’ = ActiveShore, ‘D’ = Dive, ‘I’ = Intertidal, ‘IL’ = IntertidalLand, ‘L’ = Land, ‘NR’ = NoVisualRadioOnly, ‘S’ = Shore, ‘W’ = Water); TimeDiff_min (float number of minutes between ‘from-to’); Dist (float number of meters traveled between ‘from-to’); m/hr (float the rate of travel given the measurements).
(TXT)

S3 Dataset Excel file containing all summarized data for each simulation scenario. Each tab represents the data for a specific response variable (described by the tab label). These data were extracted from the SQL Server database that contains over 2.7 billion rows of data.
(XLSX)

S1 Text Supplemental text of the individual-based model description following the “Overview”, “Design concepts” and “Details” (ODD) protocol proposed by Grimm et al. [23].
(DOCX)
