# Supplementary material for: Modeling Behavior by Coastal River Otter (Lontra Canadensis) in Response to Prey Availability in Prince William Sound, Alaska: A Spatially-Explicit Individual-Based Approach
Source: PLoS One. 2015 Jun 10;10(6):e0126208. doi: 10.1371/journal.pone.0126208 (PMC4489515; doi:10.1371/journal.pone.0126208)
Supplement: S1 Text — [23]. (DOCX) [file pone.0126208.s004.docx]

## 1. S1 Text

The individual-based model description follows the ‘Overview’, ‘Design concepts’ and ‘Details’ (ODD) protocol proposed by Grimm et al. [1]. The IBM was developed using Microsoft Visual Studio 2010 ^TM^ and Visual Basic.NET v4.0. All random values from statistical distributions were obtained using Program R via the statconnDCOM interface [2,3]. Microsoft Access ^TM^ was used to store all single-model input and output. Simulated data were then concatenated into a relational database using MS SQL Server ^TM^. All referenced tables and figures can be found within the manuscript.

### **1.1 Overview**

#### **1.1.1 Purpose**

The individual-based model (IBM) was designed to simulate the behavior and movement of coastal river otter in response to changes in prey availability within the study area using a spatially-explicit approach. Several processes directly affect the behavior and movement of otters and the subsequent variability in fecal deposition along the coastline. The processes include prey availability, abiotic habitat structure and unique social behaviors facilitating communication.

#### **1.1.2 State variables and scales**

Individual otter variables are sex, activity-state (active or resting), number of hours in current activity-state, defecation-state (defecated or not), number of hours since defecating, satiation-state (fed on pelagic fish school or not), and spatial location. For females only, the central point along the landscape network ‘edge’ of the individual’s 50% core home range, as well as the habitat encompassed by the home range, are also included as static state variables in the model. The model is constructed to track hourly activity of each individual for a period of 92 days (2208 hours), beginning on May 15, 2006 and ending August 15, 2006, matching the timeframe of field collected data. Otter movements were restricted to a landscape network comprised of 245 km of coastline and an additional 80 km of ‘virtual lines’ (Fig. 1) [4]. All movement distances and home range sizes are measured in meters.

The landscape network has three state variables assigned to each point-location, the abiotic habitat quality (likelihood of being an otter latrine), a radial-extent scaling factor, and potential pelagic fish spawning habitat. Maximum Entropy (Maxent) was used to estimate the probability of use of each 10m point-location as a latrine site (MEP) [5,6,7]. The radial-extent scaling factor was required to accurately represent female home-range size ([Section 1.3](#_4.3._Initialization).1). Because the landscape network was highly connected (i.e. island coastlines connected to themselves, etc.), a randomly drawn home-range size, if applied as a Euclidean distance radius (½ the home-range size) onto the network, would encompass a greater extent of habitat than expected. The extent of occupied habitat exceeding the randomly drawn value varied for each 10m point-location. To account for this phenomenon, the actual extent of habitat was calculated for each point-location at seven different radii (100, 500, 1000, 2000, 4000, 6000, and 10,000 meters). The ratio between the expected and actual network distances was calculated and an exponential curve was fit for each point-location. The estimated parameters were then used to scale the randomly drawn home-range value, given the central point-location of the female otter ([Section 1.3](#_4.3._Initialization).1).

The most common pelagic fish occurring in the study area were Pacific herring (*Clupea pallasi*), Pacific capelin (*Mallotus villosus*) and sand lance (*Ammodytes hexapterus*) [8]. These fish species become available to otters during seasonal spawning migrations to the nearshore environments [9,10,11]. The preferred spawning habitats of these fishes are typically shallow coastline areas, sheltered from heavy wave action and contain submerged marine vegetation [12]. Pacific herring most commonly choose spawning areas < 10m in depth [12,13]. However, depths of < 3m have been suggested as most preferable by these fish [14]. Additionally, sand lance tend to spawn in fine-grained substrate at depths of approximately 1.6m [15]. Thus, using the depth at 25m radii (Maxent variable), point-locations having a value ≥ -3.04m were included as potential spawning habitat for pelagic fish during simulations (8,108 out of 24,520 locations).

The temporal scale for fish schools was treated slightly differently than the scale of otters. Fish schools were randomly placed onto the network for each simulated hour. The assignment of school locations was restricted to the point-locations meeting the depth requirement (unless otherwise specified). However, the number of schools available during a given time step was calculated at the beginning of each 24 hour period (i.e. once per day) and held static for the entire day.

#### **1.1.3 Process overview and scheduling**

The model simulates individual otter movement and behavior through foraging, recuperation and olfactory communication processes (Fig. 2). The model begins at midnight, May 15, 2006, with each otter having predetermined states. During a given time step, the activity-state of each otter was assessed. The majority of simulated behaviors occur for otters that are currently active. Active otter’s foraging behavior is influenced by the presence of otter feces and/or fish schools. The completed foraging event is followed by defecation behavior that is driven by the defecation-state, satiation-state and spatial location of the individual. The maintenance of an individual’s current activity-state is driven by the hours within the current activity-state and the satiation-state. Recuperating otters (i.e. resting at a static location) assess the maintenance of their current state and proceed with defecation behavior if switching to the active-state.

The placement of pelagic fish schools along the coastline also occurs on an hourly basis. Each fish school is randomly placed at an available unique point-location and remains there only for the given time step. The timing and random placement reflect the dynamic movement of the pelagic fish schools within the 1-hour window [16]. The number of fish schools is calculated at 12:00am for each day of the simulation. This value is held constant for the 24-hour period.

### **1.2. Design concepts**

#### **1.2.1 Emergence**

There are two system-level patterns that emerge from the otter behavior model. The first, and primary objective of this modeling exercise, is to assess the spatio-temporal distribution of fecal deposits by otters. The resulting patterns will describe if the simulated nutrient deposition by otters are randomly occurring, creating homogenous nutrient input to the coastal environment, or if a heterogeneous pattern arises. A heterogeneous distribution pattern of nutrient deposition would create an influx of nutrients to specific locations along the coastline and influence landscape heterogeneity [17,18].

The social behavior of coastal river otters has been well documented [8,17,19,20]. Social otters use scent-markings to communicate group association while solitary otters mark territorial boundaries [17]. Thus, a second emergent pattern may arise from otter defecation behavior and communication through scent (feces) markings. The aggregation of virtual otters into social groups because of food dispersion would follow field observed social behavior and potentially explain some the heterogeneous fecal deposition patterns observed through field collected data.

#### **1.2.2 Adaptation**

The model allows for each otter, while in an active-state, to directly adapt to their current surroundings. First, foraging otters use a 50m visual-sense to locate fish schools and/or best available habitat conditions. This relatively short distance was chosen because otters are adapted to forage underwater, and are therefore nearsighted [21]. If a fish school is located, the otter stops moving along the network to feed on the school for the remainder of the time step. In addition to using sight, male otters use olfaction to detect potential prey availability. While traversing the landscape network on a foraging excursion, males have the chance to detect scent-markings left by other otters. If scent is detected, the male chooses to move in the direction of the detected scent in hopes of finding high quality prey.

#### **1.2.3 Fitness**

The defecation behavior of each otter is directly related to the type of prey consumed. Pelagic fish have higher lipid content, providing greater caloric value than demersal fish [22]. Additionally, the schooling behavior of pelagic fish provides a greater density of prey per unit area. Therefore, otters locating and feeding on a fish school increase their own fitness and potentially that of other nearby foraging otters. The individual otter locating a fish school gains additional energy per hour of foraging and increases the likelihood of reaching satiation. A more satiated otter is less likely to be in an active-state; thus hypothetically reducing the risk of predation (mortality is not included in this model) or injury through foraging excursions. Concurrently, an otter feeding on a fish school is more likely to defecate after feeding. The defecation behavior of the individual otter, coupled with other males’ attraction to scent-markings, increases the likelihood of multiple otters congregating in the location of the fish school. A group of otters have higher efficiency in capturing pelagic fish than solitary otters through cooperative foraging [8].

#### **1.2.4 Prediction**

As described in [Section 1.2.2](#_4.2.2_Adaptation), otters use detection of scent-markings as an indicator of high-quality prey availability [23]. The assumption, and subsequent prediction, made by each otter is that if a conspecific defecates, pelagic fish schools must be nearby.

#### **1.2.5 Sensing**

Each otter uses three separate types of sense: olfactory, visual and memory. The male otter uses olfaction to imperfectly detect scent-markings from other otters. The otter can smell a scent-marking up to 1km from its current location. This type of sense directly influences the choice of movement direction along the landscape network. The otter continually uses its visual sense during foraging excursions. It was assumed an otter would be acutely aware of the biotic and abiotic conditions within 50m of its current location, allowing the otter to sense nearby fish schools and the highest quality habitat. Finally, otters were assumed to have perfect memory of the local habitat. M. Ben-David has observed (personal observation) telemetered otters foraging on pelagic fish schools. In these observations, upon completion of the feeding, the otters often travelled long distances to a preferred latrine site usually with a well-established den to rest. Thus, a virtual otter transitioning from active-state to inactive-state will choose the point-location on the network having the highest habitat score (probability of being a latrine; MEP) within 1km of its current location.

#### **1.2.6 Interaction**

Individual otters are assumed to cooperatively forage during active-state interactions. It is possible for two otters to cross paths and not forage together, but only if a fish school is not present. Female otters were assigned exclusive 50% core home-ranges during model initialization [20]. However, during foraging excursions females can cross into another female otter’s core area without repercussion.

#### **1.2.7 Stochasticity**

Stochasticity was introduced into the model through four separate otter-specific processes and the behavioral parameters were expressed as probabilities or drawn from empirical statistical distributions:

1. The movement distance for an individual foraging excursion was randomly drawn from a truncated normal distribution (distance > 0) using a mean (SD) derived from 8 telemetered otters within the study area (Table 2; S2 Dataset). M. Ben-David and colleagues measured the behaviors of otters to the nearest second, for a 24 hour period for a full summer in 1991.
2. At each landscape network node, the next path chosen by a male otter was first assessed through the detection of scent-markings. For the nearest fecal deposit to the otter’s current location, a Bernoulli trial was conducted to determine if the scent was detected. The probability of successful detection was derived from a pair of equations combining the effect of distance and age of the fecal deposit (see eq. 5, 6). If no scent was detected, the next movement-path was randomly drawn from all possible paths, excluding the previously traveled path.
3. Having a successful defecation event by an otter was determined using a Bernoulli trial in which the probability was derived using a set of equations using the number of hours since the previous defecation event (mean (SD)) and the habitat quality of the currently occupied point-location as parameters (MEP; see eq. 1, 2).
4. For an otter currently in the active-state, the transition probability is derived from the mean (SD) of the timed otter behavior from the data collected for 8 telemetered otters in 1991 (S1 Dataset; see eq. 3) and tested using a Bernoulli trial.
5. The final stochastic process occurs when an otter is currently in the inactive-state. The probability of transitioning to the active-state is derived from the mean (SD) of the timed otter behavior from the data collected for 8 telemetered otters in 1991 (Table 2; S1 Dataset; see eq.4) and tested using a Bernoulli trial.
6. An additional stochastic process is the random placement of fish schools onto available point-locations. To perform this operation, the list of available potential spawning habitat point-locations was compiled with all having equal weight. Each fish school is placed onto the network, without replacement of the point-location, for each time step.

#### **1.2.8 Collectives**

Otters are known to create social groups to facilitate foraging success [8,17]. This behavior is not explicitly represented within the model. However, the use of scent-markings as an adaptive behavior by male otters may produce social collectives of otters through emergent behavior (Sections [1.2.1](#_4.2.1_Emergence) – [1.2.3](#_4.2.3_Fitness)).

#### **1.2.9 Observation**

All data output were stored within five MS Access database tables. The ‘tblOtter’ table stores otter specific information, including the otter’s sex and randomly assigned home range size (females only). The table ‘tblOtter’ relates to tables ‘tblStateValues,’ and ‘xrefHomeRange’. The table ‘xrefHomeRange’ stores the route events describing each female’s 50% core home range that cannot overlap with another female. The individual information for each otter’s state-value (time step, spatial location, activity-state, hours of activity-state, defecation-state, hours since defecating, and satiation-state) are stored in the ‘tblStateValues’ table. The spatial location and time step of each fecal deposition event is tracked in ‘tblFecesTracker’, while the timing and location of each fish school is managed in ‘tblFishSchools’ table.

The capture of information at the finest temporal scale allows for the synthesis of model results at hourly, daily, monthly or the entire study period time-scales. The temporal scales can be used to assess otter behavior for individuals, groups or the entire population. The information contained within the database can be used to perform multiple analyses. Fecal deposition rates and the spatial configuration can be described for the landscape network. On an individual basis, defecation frequency, active-state frequency, satiation-state frequency and social grouping patterns can be extracted from the data.

### **1.3. Initialization**

Portions of the landscape network were initially attributed as either within or outside the study area (Fig. 1). The study area comprised 58% of the total available coastline within the landscape network. Thus, estimates of otter demographic parameters within the study area were extrapolated to the entire landscape network.

#### **1.3.1 Otters**

The density of otters within the study area was previously estimated to range between 0.28 to 0.8 otters/km of coastline (40-115 otters) [19]. Recent abundance estimates, conducted by Ott [24], are within previous estimates and have a 95% CI equal to 55-78 otters. To begin each simulation, a value was randomly drawn from a uniform distribution bounded by Ott [24] 95% CI, representing the number of otters within the study area. The total was calculated by adding individuals to the resident population by multiplying the randomly drawn abundance by 0.585, which represents the fraction of coastline outside the study area.

The state of each otter was also determined prior to the simulation. First, each otter was assigned a sex based on an average M:F ratio (69% male) derived from previous studies [21,24,25,26]. The activity-state was assigned using a Bernoulli trial with the probability of being ‘Active’ calculated as the ratio of average hours Active:Inactive (1.43:11.69), which was equal to 0.122 (S1 Dataset). The number of hours at the current activity-state was randomly assigned using a uniform distribution bounded by 0 and the upper 95% CI for each activity-state, rounded to the nearest integer. The upper 95% CI for ‘Active’ was 1.88 hours and 15.32 hours for ‘Inactive’ ([Table](#_Table_4.1._Model) 2; S1 Dataset). Ormseth and Ben-David [27] found captive otters to defecate, on average once every 4.865 hours. Thus, the number of hours since last defecating was randomly drawn from a uniform distribution bounded by 0 and 5.

The final initialization step for each otter was the placement onto the landscape network. This step was performed separately for the study area and out-of-area portions of the landscape network. Point-locations were filtered to include only habitat values > 0.464 (MEP). This optimal threshold value for the MEP was chosen using methods described in Albeke [7]. Males were randomly assigned a point-location within the landscape network and potential overlap of locations was allowed. On the other hand, the placement of females onto the landscape network required the individual be first assigned a core home range size. Blundell et al. [20] estimated mean 50% core female home range as 4km (SD= 2) of coastline. Using these parameter values, the home range was randomly drawn from a truncated normal distribution (value > 0). Next, a random point-location was chosen and the home range size was adjusted using the radial-scale adjustment equation (Section 1.1.2) for the unique point-location. Next, the home range extent was placed onto the network by dividing the adjusted value in half and radiating out in all available paths for the remaining distance (e.g. 100m home range would be 50m in both directions). Previous studies have indicated that female 50% core home ranges do not overlap [20,28]. Thus, if any portion of the generated home range overlapped with an existing female otter, a new point-location was randomly chosen and the process repeated until all females were placed onto the landscape network.

#### **1.3.2 Pelagic Fish Schools**

The timing of the spawning migration of pelagic fish to the nearshore environment varies annually by species and magnitude [8,9,11,12,16]. Because pelagic fish schools have been shown to play an integral role in otter behavior [8,17], accounting for the timing and abundance of fish schools is important. In this model, we do not differentiate between fish school species. Rather the presence of a fish school was deemed most important.

Using georeferenced, aerially identified fish school data provided by Ben-David et al. [17], the number of fish schools within 100m of the coastline, during a one-day period, were counted for years 1996-1999. The minimum (40) and maximum (98) number of schools became the bounds for a uniform distribution used to set the maximum number of schools that could be available during a simulation. The timing of fish schools entering the simulation is explained by eq. 7.

### **1.4. Input**

The coastline portion of the landscape network was digitized from IKONOS® 1m panchromatic satellite imagery at a 1:1,500 scale, resulting in 245.3 km of coastline (Fig. 1). An additional 80.3 km of ‘virtual lines’ were appended to the network [4] to act as travel corridors connecting individual islands, or the same island having a large bay, to each other. The virtual lines were constructed through a multiple step process. First, Thiessen polygons were created using the 10m point-locations. The polygons were then converted into a line feature class. For approximately every 5 km of coastline, the line connecting two islands or a bay was retained and the excess removed. The remaining virtual lines were slightly modified to create a straight-line having only two vertices connecting the coastline(s). The Network Analyst extension within ArcGIS (ESRI, Redlands, CA) was used to identify network nodes and populate the adjacency table describing the connectivity of network edges.

### **1.5. Submodels**

For each submodel, please review Table 2 for parameter values used for each statistical distribution.

#### **1.5.1 Defecation**

The probability of an otter defecating during a given time step is driven by three factors, hours since previous defecation event, habitat quality (MEP), and satiation-state. Using the equation:

*P(de)* = ${(cdf\left( hr \right)}^{h})*s$ (1)

where the probability of a defecation event (*de*) was equal to the cumulative probability of a normal function (*cdf*) given the hours since the previous defecation (*hr*) raised to the *h^th^* (eq. 2) power and multiplied by the satiation-state (*s*). The *cdf* was parameterized using data obtained from Ormseth and Ben-David [27] and the satiation-state was equal to 2 if the otter had fed on a fish school and 1 otherwise. The *h^th^* power is equal to:

$h=-ln\left( MEP \right)*1.5$ (2)

For otters having fed on a fish school (*s* = 2), the probability of a defecation event doubles. The value of *P(de)* was used as the probability of successfully experiencing a defecation event using a Bernoulli trial. For instances when the satiation-state (*s*) increased *P(de)* to become > 1, the value was set equal to 1.

#### **1.5.2 Active-to-Inactive**

The probability of an otter transitioning from an active state to an inactive state is calculated by obtaining the cumulative probability of a normal function given the hours of being in the active-state (*cdf(hr)*) and the satiation-state (*s*):

*P(ai)* = ${cdf\left( hr \right)}^{1/s}$ (3)

The *cdf* was parameterized using the unpublished telemetry data collected by M. Ben-David. It was assumed that an otter having fed on higher quality prey would be more satiated and require less foraging effort. Thus, the satiation-state was equal to 2 if the otter had fed on a fish school and 1 otherwise. The probability of successfully transitioning to the inactive-state was tested using a Bernoulli trial. The satiation-state was reset to indicate the otter has not fed on a fish school during the following time step if the otter chose to remain active.

#### **1.5.3 Inactive-to-Active**

The probability of an otter transitioning from an inactive state to an active state is calculated by obtaining the cumulative probability of a normal function given the hours of being in the inactive-state (*cdf(hr)*) and the satiation-state (*s*):

*P(ia)* = ${cdf\left( hr \right)}^{1/s}$ (4)

The *cdf* was parameterized using the unpublished telemetry data collected by M. Ben-David. It was assumed that an otter having fed on higher quality prey would be more satiated and be more apt to remain inactive. Thus, the satiation-state was equal to 1 if the otter had fed on a fish school and 2 otherwise. The probability of successfully transitioning to the active-state was tested using a Bernoulli trial. The satiation-state remained constant for the entirety of the otter’s resting period and was reset to indicate the otter has not fed on a fish school once transitioning to the active-state.

#### **1.5.4 Male Movement - Scent Detection**

The movement of males within the landscape network is influenced by the detection of scent-markings. The probability of an otter detecting a scent-mark is driven by the distance between the otter and scent-mark and the age (hours) of the scent-mark. It was assumed that a male otter could detect another otter’s scent-mark from a distance up to 1km. This assumption is represented by the equation:

$P\left( scent \right)={Exp(-0.003*m)}^{D}$ (5)

where the probability of detecting a scent-mark (*P(scent)*) is negatively related to the distance between the scent-mark and the otter (*m*), in meters, and the age (*D*; eq. 6). Ott [24] and Guertin et al. [29] have indicated viable otter fecal samples must not be older than 24 hours because of weathering and desiccation. Therefore, to model the effect of desiccation reducing the amount of ‘scent’ available for detection, the equation:

$D= Exp(0.1*hr)$ (6)

estimates fecal desiccation (*D*) as positively related to the number of hours (*hr*) the scent-mark has been exposed to the environment. In the simulations, fecal deposits older than 12 hours were removed as possible candidates for detection. The value of eq. 5 was used as the probability of successfully detecting the scent-mark in a Bernoulli trial. It is important to note that male otters beginning their foraging excursion will seek (olfaction) scent-markings within 1km of their location in all possible directions. However, for subsequent olfaction events (at network nodes) the otter can only sense in the forward direction, not backward. Additionally, only the scent-mark having the shortest distance to the otter’s current location was tested for detection. Finally, for the current time step, all scent-marks occurring along the path traveled by the otter were removed from the list of available fecal deposits for the individual otter. If an otter did not detect a scent-mark, a random direction was chosen from a uniform distribution of all possible paths, excluding the previously traveled path (i.e. cannot move backwards, only forward).

#### **1.5.5 Female Movement - Core Home Range**

For female otters, scent-detection is not included as part of their behavior. Instead, the movement of female otters is random when occurring within the 50% core home range. The direction is randomly chosen from a uniform distribution of all possible paths, excluding the previously traveled path. However, if a movement direction and distance results in the female reaching a node or point-location outside the 50% core area, the following movement direction will be toward the central point-location of the home range.

#### **1.5.6 Fish School Timing**

As previously described in Section 1.3.2, the timing of pelagic fish school spawning migrations vary. To account for this timing, a meta-analysis was conducted using two separate studies. The first study was conducted in Prince William Sound over an eight year period beginning in 1923 [30]. The study quantified the number of ‘barrels of herring’ captured by commercial fisherman. The barrels were summed for each date-range across all years and the maximum number of barrels recorded. Next, the percent of the maximum number of barrels was calculated for each date-range. The mid-point of the date-range was used as the measurement date. Concurrently, the number of herring schools was interpreted from Fig.1 [8,16] using the 1st and 15th of each month as the day of measurement. Performing the same calculation, the percent of the maximum number of schools was calculated for each date. The data were combined by calculating the average percent of the maximum for each Julian date. These data were then fit with a 5th order polynomial regression:

$Fs= J^{5}*{1.12}^{-9} + J^{4}*{-9.88}^{-7}+J^{3}*0.00034+J^{2}*-0.0564+J*4.585-145.489 (7)$

estimating the percent of the maximum number of fish schools (*Fs*) for a given day (*J*). Thus, for the given Julian day within the simulation, the number of pelagic fish schools available is calculated as:

$F(J)=Fmax*Fs$ (8)

where *Fmax* is the maximum number of randomly drawn fish schools.

LITERATURE CITED

1. Grimm V, Berger U, Bastiansen F, Eliassen S, Ginot V, et al. (2006) A standard protocol for describing individual-based and agent-based models. Ecological Modelling 198: 115-126.

2. Baier T, Neuwirth E (2007) Excel :: COM :: R. Computational Statistics 22: 91-108.

3. R Core Team (2012) R: A language and environment for statistical computing. Vienna, Austria: R Foundation for Statistical Computing.

4. Vuilleumier S, Metzger R (2006) Animal dispersal modelling: handling landscape features and related animal choices. Ecological Modelling 190: 159-170.

5. Phillips SJ, Anderson RP, Schapire RE (2006) Maximum entropy modeling of species geographic distributions. Ecological Modelling 190: 231-259.

6. Phillips S, Dudík M (2008) Modeling of species distributions with Maxent: new extensions and a comprehensive evaluation. Ecography 31: 161-175.

7. Albeke SE (2010) Influence of individual animal behavior on spatial and temporal variability in nutrient deposition. Athens, Georgia: University of Georgia. 204 p. Available: http://athenaeum.libs.uga.edu/xmlui/handle/10724/26845. Accessed 1/15/2010

8. Blundell GM, Ben-David M, Bowyer RT (2002) Sociality in river otters: cooperative foraging or reproductive strategies? Behavioral Ecology 13: 134-141.

9. Robards MD, Piatt JF, Rose GA (1999) Maturation, fecundity, and intertidal spawning of Pacific sand lance in the northern Gulf of Alaska. Journal of Fish Biology 54: 1050-1068.

10. Norcross BL, Brown ED, Foy RJ, Frandsen M, Gay SM, et al. (2001) A synthesis of the life history and ecology of juvenile Pacific herring in Prince William Sound, Alaska. Fisheries Oceanography 10: 42-57.

11. Brown ED (2002) Life history, distribution, and size structure of Pacific capelin in Prince William Sound and the northern Gulf of Alaska. ICES Journal of Marine Science 59: 983-996.

12. Haegele C, Schweigert J (1985) Distribution and characteristics of herring spawning grounds and description of spawning behavior. Canadian Journal of Fisheries and Aquatic Sciences 42: 39-55.

13. Hay DE, McCarter PB, Daniel KS, Schweigert JF (2009) Spatial diversity of Pacific herring (*Clupea pallasi*) spawning areas. ICES Journal of Marine Science 66: 1662-1666.

14. Bargmann G (1998) Forage fish management plan: a plan for managing the forage fish resources and fisheries of Washington. In: Washington Department of Fish and Wildlife, editor. Olympia, Washington: Washington Fish and Wildlife Commission. pp. 77.

15. Penttila D (2007) Marine forage fishes in Puget Sound. Seattle, Washington: Seattle District, U.W. Army Corps of Engineers. 2007-03 2007-03.

16. Brown E, Wang J, Vaughan S, Norcross B (1999) Identifying seasonal spatial scale for the ecological analysis of herring and other forage fish in Prince William Sound, Alaska. In: Ecosystem approaches for fisheries management, editor. Alaska Sea Grant College Program, AK-SG-99–01. Fairbanks, Alaska: University of Alaska-Fairbanks. pp. 499–510.

17. Ben-David M, Blundell GM, Kern JW, Maier JAK, Brown ED, et al. (2005) Communication in coastal river otters: creation of variable resource sheds for terrestrial communities. Ecology 86: 1331-1345.

18. Roe AM, Meyer CB, Nibbelink NP, Ben-David M (2010) Differential production of trees and shrubs in response to fertilization and disturbance by coastal river otters in Alaska. Ecology 91: 3177-3188.

19. Testa JW, Holleman DF, Bowyer RT, Faro JB (1994) Estimating populations of marine river otters in Prince William Sound, Alaska, using radiotracer implants. Journal of Mammalogy 75: 1021-1032.

20. Blundell GM, Bowyer RT, Ben-David M, Dean TA, Jewett SC (2000) Effects of food resources on spacing behavior of river otters: does forage abundance control home-range size? Biotelemetry 15: 325-333.

21. Chapman J, Feldhamer G (1982) Wild mammals of North America: biology, management, and economics: Johns Hopkins University Press Baltimore, MD, USA.

22. Anthony JA, Roby DD, Turco KR (2000) Lipid content and energy density of forage fishes from the northern Gulf of Alaska. Journal of Experimental Marine Biology and Ecology 248: 53-78.

23. Kruuk H, Conroy JWH, Moorhouse A (1991) Recruitment to a population of otters (*Lutra lutra*) in Shetland, in relation to fish abundance. The Journal of Applied Ecology 28: 95-101.

24. Ott KE (2009) Recolonization or local reproduction? An assessment of river otter recovery in previously-oiled areas of coastal Alaska via non-invasive genetic sampling [M.S.]. Laramie, Wyoming: University of Wyoming. Available: http://gradworks.umi.com/14/76/1476893.html. Accessed 3/1/2010

25. Lariviére S, Walton LR (1998) *Lontra canadensis*. Mammalian Species 587: 1-8.

26. Blundell GM, Ben-David M, Groves P, Bowyer RT, Geffen E (2004) Kinship and sociality in coastal river otters: are they related? Behavioral Ecology 15: 705-714.

27. Ormseth OA, Ben-David M (2000) Ingestion of crude oil: effects on digesta retention times and nutrient uptake in captive river otters. Journal of Comparative Physiology B 170: 419-428.

28. Gorman TA, Erb JD, McMillan BR, Martin DJ (2006) Space use and sociality of river otters (*Lontra canadensis*) in Minnesota. Journal of Mammalogy 87: 740-747.

29. Guertin DA, Harestad AS, Ben-David M, Drouillard KG, Elliott JE (2010) Fecal genotyping and contaminant analyses reveal variation in individual river otter exposure to localized persistent contaminants. Environmental Toxicology and Chemistry 29: 275-284.

30. Rounsefell G, Dahlgren E (1931) Fluctuations in the supply of herring (*Clupea pallasi*) in Prince William Sound, Alaska. US Department of Commerce, Bureau of Fisheries Doc 9: 263–291.
